# Phylogeographic evidence reveals multiple colonization events and a secondary contact zone in the Balkans for the *Anthriscus sylvestris* complex (Apiaceae)

**DOI:** 10.1038/s41598-025-15409-7

**Published:** 2025-08-26

**Authors:** Renata Kurzyna-Młynik, Łukasz Banasiak, Marcin Piwczyński, Paulina Trzeciak, Mergi Daba Dinka, Alexei A. Oskolski, Krzysztof Spalik

**Affiliations:** 1 Institute of Evolutionary Biology, Faculty of Biology, Biological and Chemical Research Centre, University of Warsaw, Warsaw, Poland; 2 Department of Plant Physiology and Biotechnology, Nicolaus Copernicus University in Toruń, Toruń, Poland; 3 Independent Researcher, Toruń, Poland; 4 Department of Botany and Plant Biotechnology, University of Johannesburg, Johannesburg, South Africa

**Keywords:** *Anthriscus* sect. *Cacosciadium*, Apiaceae, Phylogeography, Diversification, Mediterranean, Biogeography, Phylogenetics, Taxonomy, Plant evolution

## Abstract

The *Anthriscus sylvestris* complex (Apiaceae) exhibits a wide ecological and geographical diversity around the Mediterranean and in Central Europe. This study aims to explore its historical biogeography. Network and phylogenetic analyses were performed using the variation in two nuclear markers (nrDNA ITS and *waxy* intron) and three plastid markers (*rpoB–trnC*, *trnS–trnG*, and *psbA–trnH* intergenic spacers) assessed for 296 accessions. Nuclear and plastid markers disagreed with each other and with the current taxonomy of the complex. Two ribogroups, *Nit* and *Syl*, were apparent, with the former encompassing mountainous taxa from the Balkan Peninsula and Central Europe and the latter uniting remaining accessions. Plastid data suggested a Middle Eastern origin of the complex, with migration to Europe around the Mediterranean through the Iberian Peninsula, and a secondary contact with another migration wave in the Balkans. However, a scenario with two migration waves to Europe through the Balkans cannot be excluded. *Waxy* data distinguished a more detailed geographical and ecological pattern. The estimated age of the *A. sylvestris* complex based on the plastid data was 1.72 Ma, whereas the divergence within the European group began approximately 0.44 Ma.

## Introduction

The aridification of the climate in the Miocene and the onset of the Pleistocene Ice Age almost completely wiped out the tropical forest flora of Europe^1,2^, whose remnants were confined to the Mediterranean region and supplemented with taxa adapted to temperate and arid climates, such as Arctotertiary floristic elements or xerophytes^2^. The Ice Age severely depleted the biodiversity of Europe and the Mediterranean but also increased diversification through allopatric speciation in glacial refugia and during the onset of the Mediterranean climate. Phylogenetic studies reveal some common cladogenetic and phylogeographic patterns in the Mediterranean plants^3–5^. The same events—namely, the periods of repetitive glaciations, the Messinian salinity crisis, and the onset of the Mediterranean climate—also affected the taxa inhabiting mountain ranges in Europe and around the Mediterranean region, contributing to their diversification and circum- or amphi-Mediterranean disjunctions^6,7^. These taxa are not Mediterranean in the strict sense, i.e., they are not adapted to the Mediterranean climate, but their biogeographic history is linked to the geoclimatic changes of the Mediterranean Basin. In contrast to the strictly Mediterranean plants, the mountainous taxa in the Mediterranean Europe do not seem to exhibit a common diversification pattern^8^, pointing out to the complex biogeographic history of the region.

Among the immigrant taxa adapted to temperate and arid climates of the Pleistocene Europe and the Mediterranean region were Apiaceae subfamily Apioideae. These are mostly plants of open, often dry habitats^9^, and biogeographic reconstructions suggest that the Mediterranean and western Asia played a significant role in their diversification^10^. An intriguing example of a non-Mediterranean taxon but with the center of diversity around the Mediterranean is *Anthriscus* sect. *Cacosciadium* (Apiaceae), also identified as *A. sylvestris* (L.) Hoffm. sensu lato (s.l.) or the *Anthriscus sylvestris* complex^11–13^. It includes *A*. *nitida, A. lamprocarpa*, *A. schmalhausenii*, and a polymorphic *A. sylvestris* encompassing at least four subspecies (Table 1). *Anthriscus sylvestris* subsp. *sylvestris* is widespread and occurs from temperate Europe through the mountains of Central Asia to the Far East, and in the mountains of North Africa and the tropical East Africa. In the mountains of Southeast Europe, the Middle East, and the Irano-Turanian floristic region, it is replaced by subsp. *nemorosa*, which differs from subsp. *sylvestris* in having tuberculate bristled fruits as opposed to glabrous fruits in the former. Interestingly, the populations inhabiting the mountains of tropical East Africa are mixed with respect to fruit indumentum: although most have smooth fruits, some plants with tuberculate fruits also occur.

**Table 1:** Synopsis of the *Anthriscus sylvestris* complex (*Anthriscus* sect. *Cacosciadium*).

Taxon name	Fruit	Habitat	Distribution
*A. nitida*	black, glabrous	Shady montane beech-fir-maple forests	Mountains of central and south-eastern Europe (excluding the Peloponnese)
*A. lamprocarpa*	straw-yellow, glabrous	Semi-open lower-montane Mediterranean-forests (maquis)	East Mediterranean: from SE Turkey to Israel
*A. schmalhausenii*	black, glabrous	Shady montane beech-hazel forests	Southern slopes of the Caucasus
*A. sylvestris* subsp. *sylvestris*	black, glabrous	Open anthropogenic habitats (meadows, roadsides etc.), semi-open forests	Widespread in Eurasia, scattered in the mountains of North and East Africa
*A. sylvestris* subsp. *alpina*	black, glabrous	Open or semi-open alpine or subalpine screes	Europe: Alps, Vosges, Jura
*A. sylvestris* subsp. *nemorosa*	brown to black, tuberculate	Forests and thickets, subalpine meadows	FSrom SE Europe to E Asia, more to the south than subsp. *sylvestris*
*A. sylvestris* subsp. *fumarioides*	brown, tuberculate	Semi-open screes subalpine screes	Europe: NW Balkan Peninsula

In the eastern Mediterranean (SE Turkey, Syria, Lebanon, Jordan, and Israel), subsp. *nemorosa* is replaced by *Anthriscus lamprocarpa* that has glabrous fruits. These two taxa are parapatric and may have evolved from a common ancestor through geographic isolation. Alternatively, *A. lamprocarpa* may have originated from North African populations of *A. sylvestris* subsp. *sylvestris* (once placed in a separate subspecies *mollis*), as these show some similarity in habit^11^, However, at present there is a considerable geographical gap between these two taxa. In contrast, the European endemic taxa—*A. nitida*, *A. sylvestris* subsp. *fumarioides*, and *A. sylvestris* subsp. *alpina*—are sympatric with *A. sylvestris* subsp. *sylvestris*, but they are ecologically differentiated. This suggests the importance of ecological rather than geographical barriers in their diversification. A molecular phylogenetic study based on nrDNA ITS sequence variation confirmed the monophyly of the *A. sylvestris* group but suggested that the widely distributed subsp. *sylvestris* is paraphyletic, albeit with a very low internal support^14^.

In Europe, *A. sylvestris* subsp. *sylvestris* typically thrives in lowland habitats, primarily in riparian forest, but most frequently occupying anthropogenic environments such as wet meadows and roadsides. In contrast, *A. nitida* is primarily found within the beech-forest belt of the Central and Southeast European mountains, spanning from the Alps and Vosges to the Carpathians and Balkans. Within the Alps and Vosges, it coexists with *A. sylvestris* subsp. *alpina*, which occurs in open or partially shaded subalpine screes. Both taxa exhibit glabrous fruits, distinguishing them from *A. sylvestris* subsp. *fumarioides*, which possesses tuberculate fruits and is found in partially shaded subalpine screes within the western Balkan Peninsula mountains, akin to the habitats of subsp. *alpina*. This extensive ecological range of the *A. sylvestris* complex, encompassing lowland to subalpine locations and spanning open anthropogenic environments to mature shaded forests, is unparalleled among any other umbellifer species or species complexes found in Europe^11,15^. Questions arise about the origin of this diversity and the relationships among the taxa included, particularly of the narrow endemics in relation to the widely distributed subspecies *sylvestris* and *nemorosa*. Former biogeographic analyses suggested that the tribe Scandiceae and most of its genera, including *Anthriscus*, originated in the Middle East^10^, and spread from there to Europe and the rest of Asia. The currently recognized taxa may have therefore originated through geographic isolation (allopatric speciation), particularly in the glacial refugia in Europe and the Caucasus. However, an important question is whether these taxa—defined as morphotypes and ecotypes—are indeed genetically differentiated.

There is growing evidence for the role of a “combinatorial” mechanism of speciation: old genetic variation, previously tested by selection and occurring at higher allele frequency than new mutations, may speed up speciation and facilitate adaptive evolution^16^. These old genetic variants may be obtained from conspecific, formerly isolated populations or from closely related species due to hybridization and introgression. Therefore, an additional question is whether central and southeastern Europe, the area with the highest diversity of the *A. sylvestris* group, might have also been the place of secondary contact between two migration waves, spurring the ecological and morphological diversification.

The *A. sylvestris* complex occurs around the Mediterranean, raising the questions of what the migration routes were and whether the distribution ring closed. It has been suggested that *A. lamprocarpa* originated from North African populations of *A. sylvestris* subsp. *sylvestris*^11,12^, implying that the ring closed in the Levant, with *A. lamprocarpa* and *A. sylvestris* subsp. *nemorosa* representing the most genetically distant populations. The drawback of this hypothesis is the significant distribution gap between *A. lamprocarpa* and the North African populations of *A. sylvestris*, as this taxon is absent from Libya and Egypt. However, these taxa may have been present in this region in the past, when the climate was cooler and more humid. An alternative hypothesis tested in this study is that the Balkans served as a secondary contact zone for divergent lineages within the *Anthriscus sylvestris* complex. The presence of morphologically and ecologically distinct forms in this region suggests that range overlap and lineage mixing may have driven the diversification of montane *A. nitida* and *A. sylvestris* subsp. *fumarioides*, alongside lowland or low-montane *A. sylvestris* subsp. *sylvestris* and subsp. *nemorosa*. The coexistence of *A. nitida* and *A. sylvestris* subsp. *sylvestris* in the Carpathians, the Balkans, and the Alps further raises questions about their genetic distinctiveness.

This study aims to explore the historical biogeography of the *Anthriscus sylvestris* complex in order to infer its origin and migration routes, and, in particular, to identify possible areas of secondary contact between migration waves that may have contributed to the diversification of this taxon.

## Materials and methods

### Molecular markers

Phylogenetic relationships were inferred from two nuclear markers—rDNA ITS and *waxy*—and three plastid (pDNA) intergenic spacers: *rpoB–trnC*, *trnS–trnG* and *psbA–trnH*. The nuclear ribosomal DNA internal transcribed spacer has previously been successfully used in phylogenetic analyses of umbellifers, both at the subfamily Apioideae level^17^ and at the infrageneric level^18,19^. The granule-bound starch synthase I gene—GBSSI or *waxy*—is a single-copy nuclear gene that appears to be more phylogenetically informative than ITS at the infrageneric level^20^. This marker has not yet been used in phylogenetic studies of apioid umbellifers. The intergenic spacers *rpoB–trnC*, *trnS–trnG*, and *psbA–trnH* are among the most variable and commonly used plastid markers^21,22^. They were also applied in phylogenetic studies of umbellifers at the infrageneric level^23–25^.

### Taxon identification and sampling

**Fig. 1:**
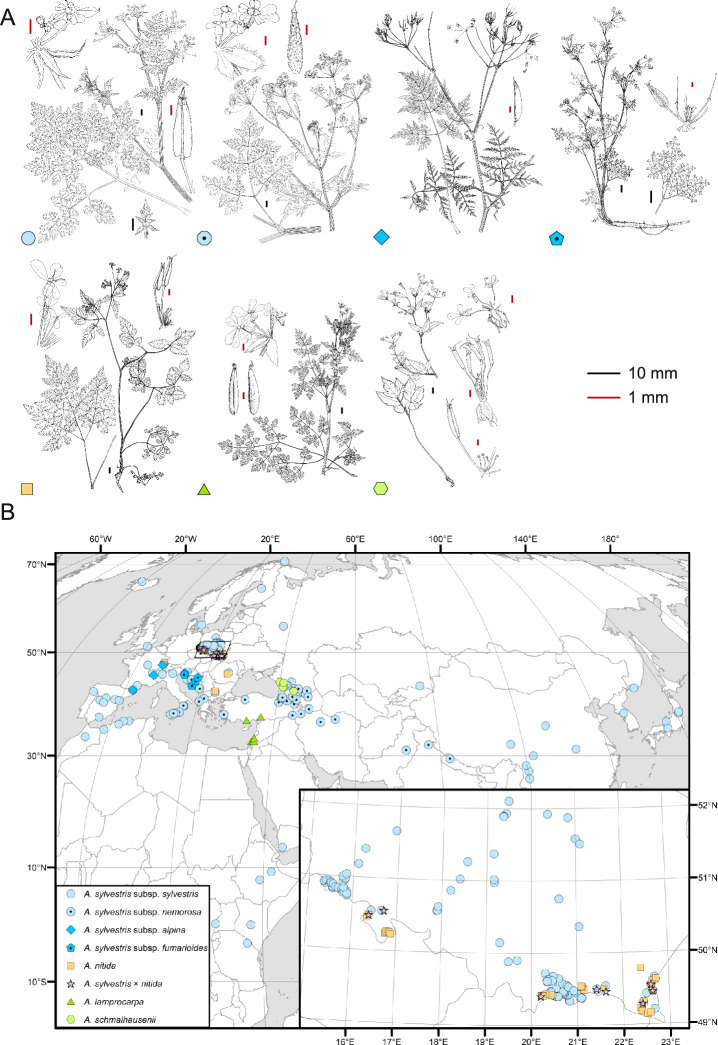
(**a**) Species and subspecies of *Anthriscus* sect. *Cacosciadium* considered in this study. (**b**) Location of plant samples. Shapes denote respective taxa (see the legend). Shapes with dots denote plants with bristled ovaries or fruits. Inset presents dense sampling of mountainous *A. nitida* and lowland *A. sylvestris* subsp. *sylvestris* in their contact zone in southern Poland.

We paid attention to two key morphological markers crucial for distinguishing sympatric taxa: fruit tuberculation and basal (or lower cauline) leaf size^11^. Small tubercles ending in short bristles that cover the entire fruit are distinguishing features of *A. sylvestris* subsp. *nemorosa* and subsp. *fumarioides*, setting them apart from sympatric or parapatric *A. sylvestris* subsp. *sylvestris*, *A. lamprocarpa*, and *A. schmalhausenii* (Fig. 1a). These bristles are also visible on ovaries, enabling identification of these taxa during the flowering stage.

The size of the primary leaf division helps distinguish *A. nitida* from *A. sylvestris* subsp. *sylvestris*. If it is similar to the rest of the blade (i.e., the leaf is tripartite rather than pinnatisect), then the specimen is likely to be *A. nitida.* In contrast, if the primary division is distinctly smaller than the rest of the blade (i.e., the leaf is distinctly pinnatisect rather than tripartite), then the plant may be identified as *A. sylvestris* subsp. *sylvestris*.

Additionally, we considered the presence of short bristles or scales at the base of the fruit (present in *A. sylvestris* subsp. *sylvestris*, absent in *A. nitida*) and the length of pedicels (pedicels longer than fruits in *A. nitida*, pedicels shorter than fruits in *A. sylvestris* subsp. *sylvestris*). Specimens displaying intermediate characteristics collected in a potential suture zone in the Carpathians were identified as hybrids, *A. sylvestris* × *A. nitida*.

In total, 296 accessions were sampled from herbarium collections and from the wild (voucher specimens are listed in Table S1, the plant material was used in accordance with all relevant guidelines and legislation.). The outgroup to *Anthriscus* included three species of *Kozlovia*, a genus identified as its sister in previous molecular studies^14^. To provide proper rooting of sect. *Cacosciadium*, all species of the other sections of the genus were also considered, each represented by at least two accessions. The *A. sylvestris* complex included 273 samples representing all recognized taxa and covering its entire natural range in Eurasia, North Africa and mountains of tropical East Africa^11^ (Fig. 1b). Because our preliminary results indicated occurrence of different ribotypes (nrDNA ITS) in mountainous *A. nitida* and lowland *A. sylvestris* subsp. *sylvestris*, we densely sampled these taxa from the wild in the Polish Carpathians and the Sudeten Mountains (inset in Fig. 1b). As the preliminary results indicated a low resolution of plastid data for the European taxa, these additional samples were examined only for ITS sequence variation.

### Molecular analyses

Total genomic DNA was extracted from approximately 20 mg of plant tissue (leaves, flowers or immature fruits) using the DNeasy Plant Mini kit (Qiagen, Hilden, Germany) according to the manufacturer’s protocol. For amplification of ITS sequences, the external primers NnC18S10 and C26A were used^26^. However, due to DNA degradation in some herbarium specimens, amplifying the entire ITS region was challenging. In such cases, ITS1 and ITS2 were separately amplified using the primers 18S-ITS1-F and 5.8S-ITS1-R for ITS1 and ITS3-N and C26A for ITS2^27^, then assembled based on overlapping conserved sequence of 5.8S rDNA. All 296 samples were successfully sequenced for the ITS region. Occasional polymorphic sites were marked using IUPAC codes.

Plastid *rpoB–trnC*, *trnS–trnG*, and *psbA–trnH* intergenic spacers were amplified using standard external primers^21^. However, for some samples it was impossible to obtain the entire *rpoB–trnC* spacer, so alternative external and internal primers were designed using PrimerSelect^28^: renF (ATTCTGCTACTTAGGCTTAC), renR (AAGGATACATAACMAATCAG), 1R (TTCATTTTTCTGGTATTC), 2R (AAAAATACAACCCCTCTT), 3F (GCATACGCTAAGGATTGTG), 3R (ATTTGAACCATTAACTATTGACTC), and 4R (TTTTTAGTTTCTTGTGTCATTAG). PCR amplification and Sanger sequencing protocols are given elsewhere^24,27^. All three plastid regions were successfully obtained for 101 samples.

Due to DNA degradation in herbarium samples, amplifying the entire granule-bound starch synthase I gene was impossible. Additionally, amplifying the gene in parts and reassembling was impractical due to the presence of two different alleles in several accessions as all species of *Anthriscus sylvestris* complex are diploid with 2*n* = 16. Therefore, we chose a region covering approximately 640 bp and spanning from exon 10 to exon 13. This region was amplified using primers 10F and 13R^29^. The amplification was successful for 40 samples, but direct sequencing of the PCR products for 11 samples revealed allele polymorphisms. To resolve the sequences of these alleles, PCR products were cloned using a PCR Cloning Kit (Qiagen) and JM109 Competent Cells (Promega, Madison, Wisconsin, USA), and then sequenced. Effectively, 51 sequences were included in subsequent analyses.

Contig sequences were assembled and edited using SeqMan^28^. All sequences have been deposited in GenBank (see Table S1). For each marker, DNA sequences were aligned using mafft v. 7.271^30^, and the resulting matrix was manually corrected if necessary using Mesquite v. 3.6^31^ or AliView v. 1.28^32^. In the *psbA–trnH* intergenic spacer, a highly homoplastic inversion of 12 base pairs occurred in several accessions. This inversion is common in many species of umbellifers at infraspecific level^33^. Therefore, it was excluded from phylogenetic analyses.

Voucher data and GenBank accession numbers are given in Table S1. Matrices and trees were deposited in the University of Warsaw repository danebadawcze.uw.pl, 10.58132/WACYXJ .

### Network, phylogenetic and phylogeographic analyses

The congruence of molecular markers was assessed in two datasets: a 101-accession dataset (pDNA + ITS), and a 51-accession dataset (pDNA + ITS + partial waxy gene). This evaluation was conducted using a hierarchical likelihood ratio test, as implemented in Concaterpillar v. 1.7.2^34^. To accommodate the heterozygosity observed in 11 samples for the waxy gene, the pDNA and ITS data were duplicated for each respective sample. Characteristics of the datasets are provided in Supplementary Table S2.

Haplotype networks were constructed using TCS method^35^, as implemented in POPART v. 1.7^36^. For these analyses, polymorphic sites were resolved to minimize branch lengths, whereas sites with indels were excluded. As the outgroup, we only used the species of *Anthriscus*, because the inclusion of *Kozlovia* did not impact the relationships within *A. sylvestris* s.l. while, due to the long branches, made it challenging to visualize the networks.

Phylogenetic trees were inferred using both maximum likelihood (ML) and Bayesian inference (BI) methods. Each group of identical sequences was represented by a single terminal in the analyses. ML analyses were conducted using RAxML v. 8.2.4^37^, utilizing the GTR + G nucleotide substitution model, the only model available in this program. Tree internal support was estimated through 1000 rapid bootstrap replicates.

The optimal nucleotide substitution models for BI analyses were determined for each dataset using PartitionFinder2^38^ utilizing BIC criterion. BI trees were generated using a parallel version of MrBayes v. 3.2.6^39^ with default priors. The analyses involved two independent runs of four Monte Carlo Markov chains for a total of 10 million generations, with samples taken every 1000 generations. A burn-in of 25% was applied. The convergence of runs and the effective sample size were assessed using Tracer v. 1.7.1^40^. The resulting trees were summarized into a 50% majority rule consensus tree.

Divergence times were estimated using BEAST v. 1.10.4^41^, separately for the ITS and pDNA data. To calibrate the trees, we utilized the divergence between *Kozlovia* and *Anthriscus*, previously estimated from the ITS analyses of Apioideae^10^. To implement this calibration, we assumed a normal prior distribution with a mean of 8.792265616 and a standard deviation of 1.158792094 as inferred based on the analysis of posterior sample from the previous study. This prior distribution was applied to the root of the tree.

We estimated divergence times with topological constrains enforcing *Kozlovia* and *Anthriscus* to be monophyletic taxa. In these analyses, the tree topology was estimated concurrently with the divergence times. The chain length, sampling frequency, and DNA substitution models were consistent with the parameters used in the previous MrBayes analyses.

The results of the network and phylogenetic analyses were also considered in a geographic context. All samples were georeferenced using point-radius method implemented in Georeferencing Calculator^42^. Geographic patterns were visualized with ArcGIS 10.8.2.^43^.

## Results

### Molecular markers’ congruence

Hierarchical clustering of the markers in the 101-terminal dataset concatenated *rpoB–trnC* and *trnS–trnG* spacers with *P* = 1.0, and these two with *psbA–trnH* spacer with *P* = 0.21. However, it rejected joining pDNA data and ITS data with *P* = 0.006. In the 51-terminal dataset encompassing all markers, *waxy* data were not incongruent with ITS data, but with a rather low *P* = 0.076. Therefore, in subsequent network and phylogenetic analyses, ITS, pDNA, and partial *waxy* datasets were analyzed separately.

### Haplotype network analyses

For haplotype network analyses, we used full matrices containing 296 (ITS), 101 (pDNA), and 51 (*waxy*) sequences, respectively.

**Fig. 2:**
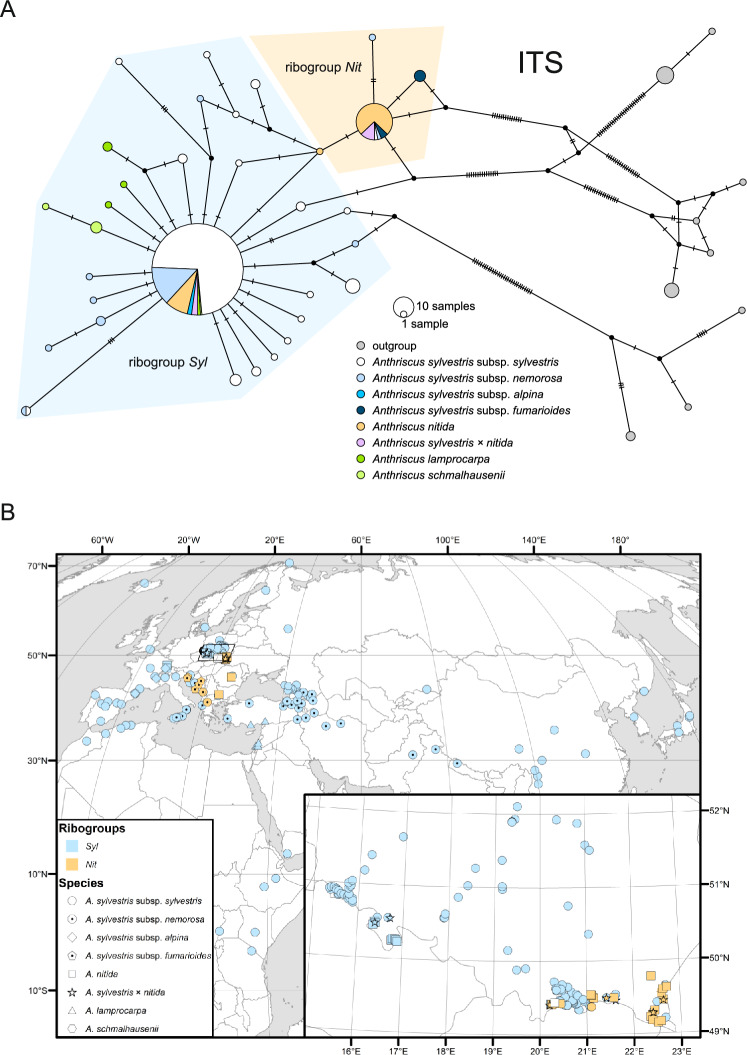
Sequence variation and geographical distribution of nrDNA ITS haplotypes of the *A. sylvestris* complex. (**a**) TCS haplotype network; pie-chart colors correspond to taxa, whereas *Syl* and *Nit* ribotype groups are indicated with tint. (**b**) Geographical distribution of *Syl* and *Nit* ribotype groups; the groups are marked with color while shapes denote taxa. The sample of *A. nitida* that provided the ribotype intermediate between both ribogroups is marked with white square.

Among the 296 ITS haplotypes (ribotypes), two ribotypes—denoted thereafter as *Syl* and *Nit* ribotypes—were most common (Fig. 2a). Ribotype *Nit* differs from ribotype *Syl* in two substitutions and one deletion (that was omitted in the network analyses), a possibly synapomorphic combination. The *Syl* ribotype and derived ribotypes form the *Syl* ribogroup, which is geographically and taxonomically widespread. In Europe, it is characteristic for lowland *A. sylvestris* subsp. *sylvestris* and mountainous subsp. *alpina* as well as for the representatives of *A. nitida* from western Europe; it also encompasses *A. lamprocarpa*, *A. schmalhausenii*, and non-European representatives of *A. sylvestris* subsp. *nemorosa* (Fig. 2b).

In the broadly defined *Syl* ribogroup, there are ribotypes that differ from the *Syl* ribotype in up to four substitutions, which is greater than the difference between the *Syl* and *Nit* ribotypes. Some of these variations are found in accessions from outside of Europe, areas that have not been studied as thoroughly as Europe. With more extensive sampling, it is possible that some of these accessions will form distinct ribogroups.

In contrast to *Syl*, the ribogroup *Nit* is geographically constrained and occurs in *A. nitida*, *A. s.* subsp. *nemorosa*, and *A. s.* subsp. *fumarioides* from the Carpathians and the Balkan Peninsula (Fig. 2b). In Poland, ribotype *Nit* is restricted to the Carpathian representatives of *A. nitida*, while ribotype *Syl* occurs in *A. s.* subsp. *sylvestris* and *A. nitida* specimens from the Sudeten Mountains (see the inset in Fig. 2b). Specimens of *A. s.* subsp. *sylvestris* with *Nit* ribotype and those of *A. nitida* with *Syl* ribotype, as well as plants with intermediate features were also sporadically found, indicating possible hybridization in the contact zone. It is noteworthy that the rooting of the network is ambiguous as the outgroups representing other species of *Anthriscus* did not form a group.

**Fig. 3:**
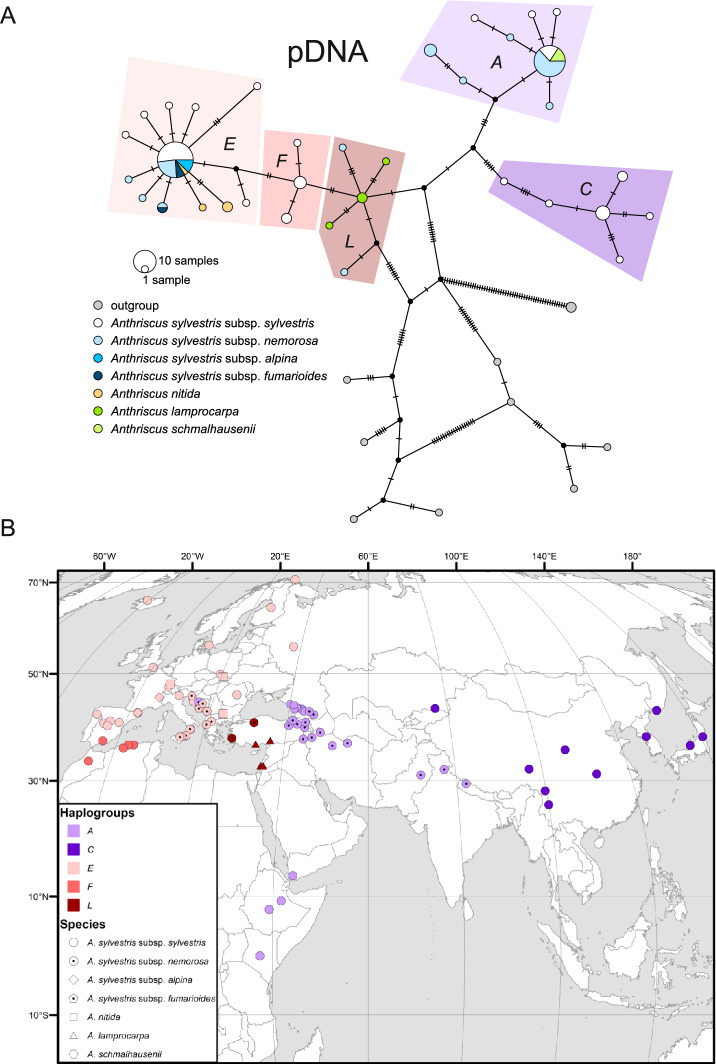
Plastid haplotype variation and geographical distribution of the *A. sylvestris* complex. (**a**) TCS haplotype network; pie-chart colors refer to taxa, whereas plastid haplotype groups are indicated with tint. (**b**) Geographical distribution of haplotype groups; the groups are marked with color and shapes identify taxa.

A strong geographical structure, rather than a taxonomical one, is evident in plastid sequence variation (Fig. 3). In the haplotype network (Fig. 3a), the central haplogroup, henceforth referred to as *L*, comprises representatives of *A. lamprocarpa* and *A. s.* subsp. *nemorosa* from the eastern Mediterranean, specifically the Levant and Asia Minor (Fig. 3b). Nearly all European samples, regardless of their taxonomical origin, fall into haplogroup *E*, which is connected to haplogroup *L* by the North African haplogroup *F*. The majority of western and central Asiatic accessions of *A. sylvestris* subsp. *sylvestris* and subsp. *nemorosa* belong to haplogroup *A*, with one accession from central Asia positioned closer to the eastern Asiatic *C* haplogroup, which unites specimens from China, the Russian Far East, and Japan. Haplogroup *A* also includes Afromontane samples, i.e., from tropical East Africa. The rooting of the *A. sylvestris* s.l. tree is, however, ambiguous: it could either indicate that haplogroup *L* was ancestral to the others, or that the primary split occurred between the western (*L* + *F* + *E*) and eastern (*A* + *C*) groups.

**Fig. 4:**
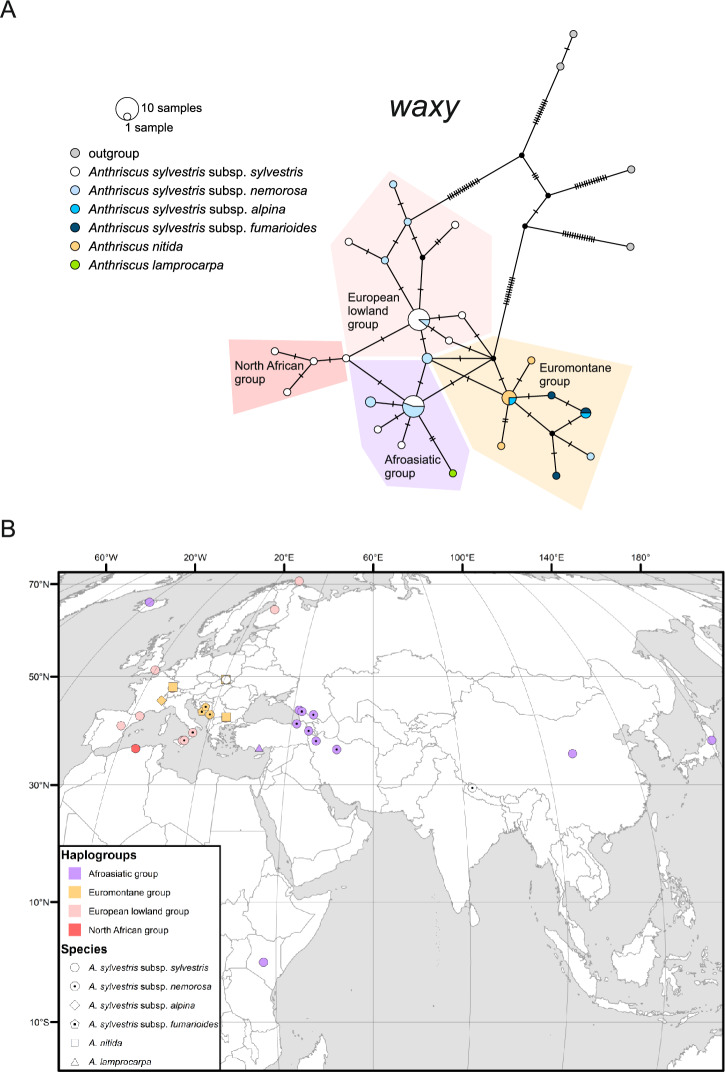
Partial *waxy* allele variation and geographical distribution of the *A. sylvestris* complex. (**a**) TCS haplotype network; pie-chart colors refer to taxa, whereas allele groups are indicated with tint. (**b**) Geographical distribution of allele groups; the groups are marked with color and shapes identify taxa. White shapes indicate intermediate haplotypes.

In the haplotype network for the *waxy* gene (Fig. 4a), similar to the ITS network, eastern and south-eastern European accessions of *A. sylvestris* subsp. *nemorosa* and subsp. *fumarioides*, along with *A. nitida* – all characterized by the *Nit* ribotype – were grouped together. However, in contrast to the ITS network, this group, hereafter designated as the Euromontane group, also included accessions of *A. nitida* and *A. sylvestris* subsp. *alpina* from the Alps, all of which had the *Syl* ribotype. The remaining European accessions, primarily consisting of plants from lower elevations, formed the European lowland group. Asiatic and mountainous accessions from the tropical East Africa constituted another haplogroup, hereafter referred to as the Afroasiatic group. North African accessions formed a branch that was equidistant from the European lowland group and from the Afroasiatic group. Therefore, the pattern of variation in the partial sequence of the *waxy* gene appears to reflect both the geography and ecology of the included accessions (Fig. 4b). The rooting of the network remains ambiguous, as the outgroup is connected either to the Afroasiatic group or the European lowland group.

### Phylogenetic analyses

Because the plastid DNA, rDNA ITS, and partial *waxy* sequences displayed incongruence in hierarchical likelihood tests as well as somewhat different geographical and ecological patterns in the network analyses, the respective datasets were subject to separate phylogenetic analyses. Additionally, each group of identical sequences was represented by a single terminal. This resulted in 58 terminals for the ITS dataset, 63 terminals for the pDNA dataset, and 32 terminals for the *waxy* dataset. PartitionFinder returned SYM + G, GTR + G and HKY + G models of nucleotide substitution for ITS, pDNA and *waxy* datasets, respectively.

The ITS trees (Fig. S1) obtained using maximum likelihood and Bayesian methods showed poor resolution. The monophyly of the *A. sylvestris* complex was strongly supported (BS = 100%; PP = 1.0) with the most of *Nit* ribotype group practically forming a basal polytomy, due to very short internal branches. Similarly to the network analyses, the relationships within the *Syl* ribotype group remained unresolved. This group, in addition to *A. s.* subsp. *sylvestris* and subsp. *nemorosa*, also included *A. s.* subsp. *alpina*, *A. nitida* from France, *A. lamprocarpa*, and *A. schmalhausenii*. Section *Cacosciadium* was identified as the sister group to the section *Anthriscus*, comprising *A. caucalis* and *A. tenerrima* (BS = 77%, PP = 0.99).

**Fig. 5:**
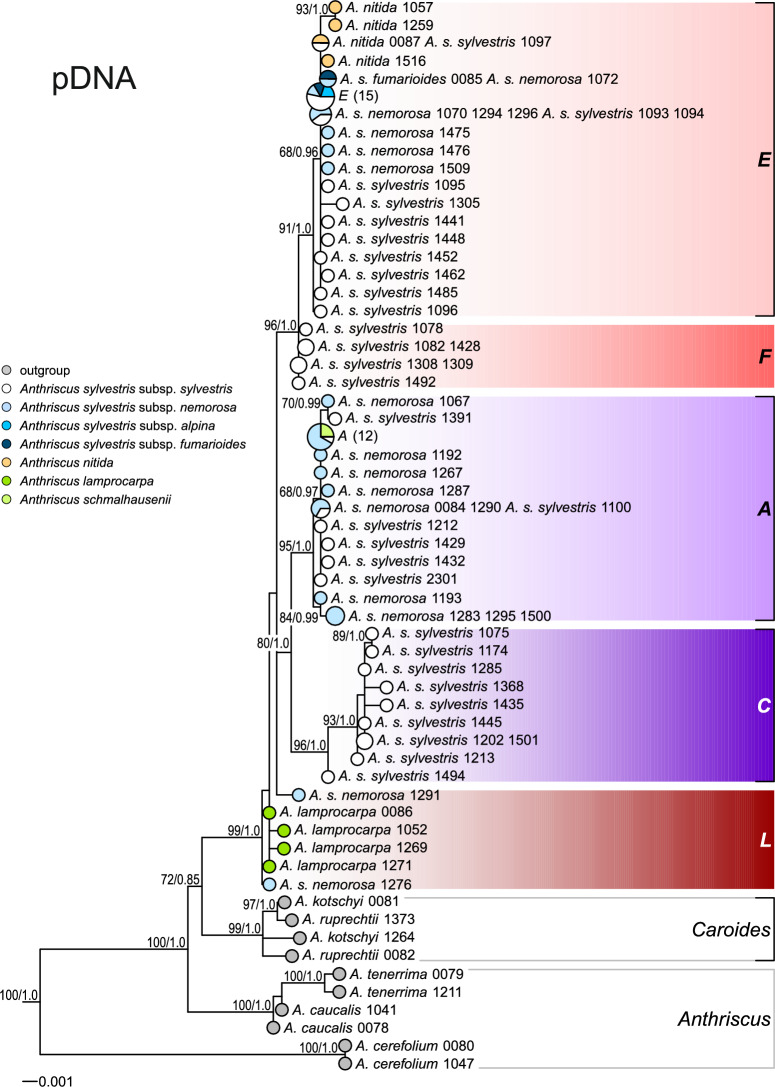
Maximum likelihood tree obtained from concatenated plastid *rpoB–trnC*, *trnS–trnG*, and *psbA–trnH* spacers. Pie-chart colors correspond to taxa, and plastid haplotype groups are indicated with tints, as in Fig. 3. Two common haplotypes in haplogroups E and A are denoted by the letters of their respective haplogroup, with the number of samples in parentheses. Bootstrap support values (> 50%) and posterior probabilities (when available, > 0.5) from additional Bayesian analyses are indicated along the branches. For simplicity, *Kozlovia* was omitted.

Analogously to the network analyses, plastid DNA trees exhibited a clear phylogeographic pattern. The root of the sect. *Cacosciadium* was different in ML and BI trees (although consistent with the network analysis: cf. Figure 3). In ML tree (Fig. 5), an accession of *A. s*. subsp. *nemorosa* from Turkey (#1276) was placed sister to the remaining representatives of the *A. sylvestris* complex, followed by *A. lamprocarpa*. In BI trees (Fig. S3), *A .s. nemorosa* #1276 was embedded, with *A. lamprocarpa*, in a trichotomy with a clade comprising the North African (F) and European accessions (E), whereas the main split was between western (*L* + *F* + *E*) and eastern (*A* + *C*) groups (cf. Figure 3a).

In the *waxy* ML trees (Fig. S2A), the North African, Euromontane, and Afroasiatic groups from the network analysis formed clades nested within the paraphyletic European lowland group. Interestingly, the Euromontane clade included all examined samples of *A. nitida*, both those carrying the *Nit* ribotype and *Syl* ribotype, thereby highlighting an ecological component of sequence variation within this molecular marker. However, in the Bayesian analyses (Fig. S2B), only the Euromontane and North African clades were retained.

It is noteworthy that various markers indicated different sister taxa to sect. *Cacosciadium*: ITS data supported its affinity to sect. *Anthriscus*, encompassing *A. caucalis* and *A. tenerrima*, whereas plastid sequences pointed to sect. *Caroides*, consisting of *A. kotschyi* and *A. ruprechtii*, albeit with a moderate internal support (BS = 72%; PP = 0.85).

In all trees except for the ML plastid tree, *A. caucalis* and *A. tenerrima* were identified as sister taxa, whereas the accessions of *A. kotschyi* and *A. ruprechtii* were always intermingled.

### Estimating divergence times

**Fig. 6:**
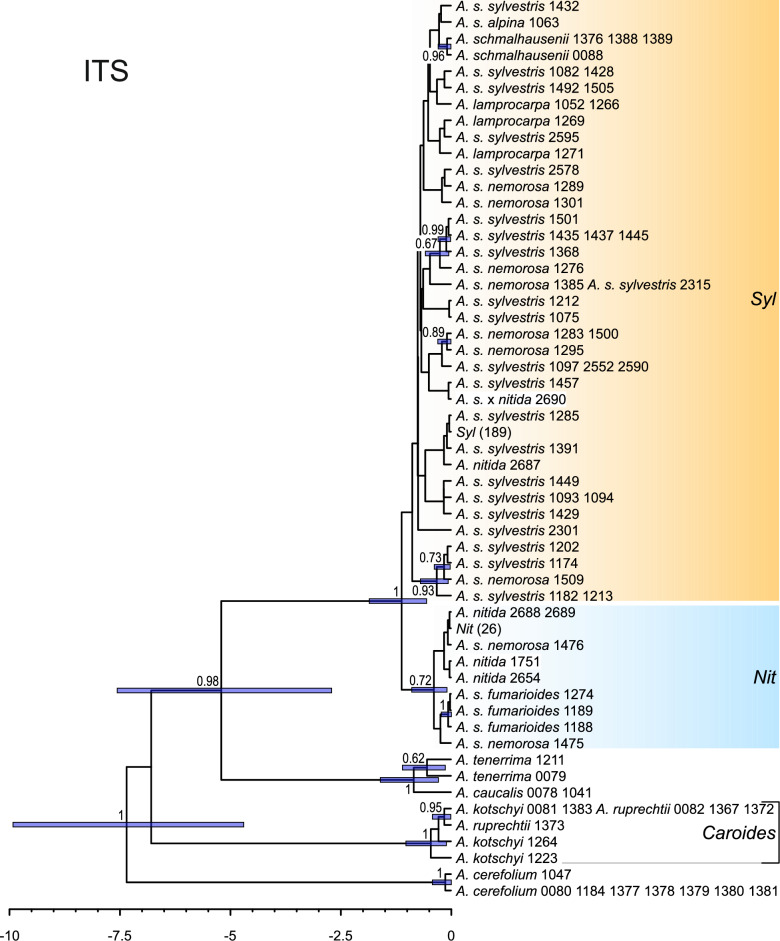
Chronogram obtained with BEAST from ITS data constraining the root of the tree, i.e. specifying *Kozlovia* and *Anthriscus* to be each monophyletic. The root was assumed to have a normal distribution with a mean of 8.792265616 and a standard deviation of 1.158792094, based on the analyses of Banasiak, et al. ^10^. For simplicity, *Kozlovia* was omitted. Horizontal bars denote 95% HPD intervals; PP values (> 0.5) are provided along branches. Ribogroups are the same as in Fig. 2.

In BEAST analyses using the ITS data, the most recent common ancestor of the *A. sylvestris* complex was estimated to live 1.13 million years ago (Ma), with a 95% highest posterior density (HPD) interval ranging from 1.86 to 0.56 Ma (Fig. 6). It is noteworthy that the ribotype groups *Nit* and *Syl* were sisters, rather than the latter being nested within the former (compare with Fig. S1). However, both clades received a very low PP support: 0.36 and 0.72 for monophyly of *Syl* and *Nit*, respectively. The *Nit* group began to diversify approximately 0.40 Ma, whereas the initial split in the *Syl* group occurred at 0.89 Ma.

**Fig. 7:**
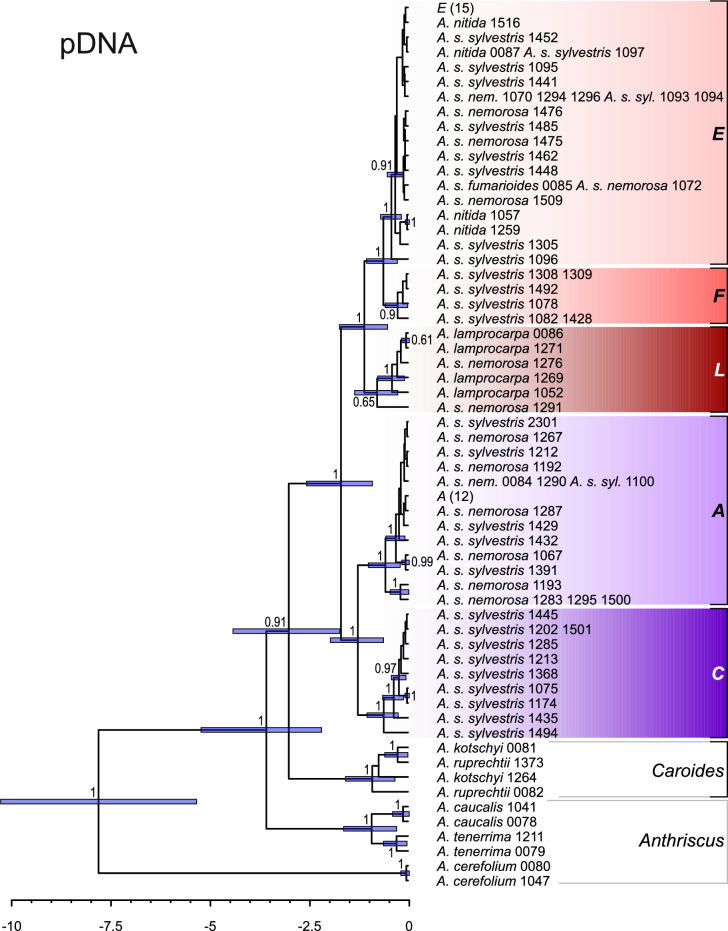
Chronogram obtained with BEAST from concatenated plastid *rpoB–trnC*, *trnS–trnG*, and these *psbA–trnH* spacers with the constraint on the root of the tree, i.e. specifying *Kozlovia* and *Anthriscus* to be each monophyletic. The root was assumed to have a normal distribution with a mean of 8.792265616 and a standard deviation of 1.158792094, based on the analyses of Banasiak, et al. ^10^. For simplicity, *Kozlovia* was omitted. Horizontal bars denote 95% HPD intervals; PP values (> 0.5) are provided along branches. Plastid haplogroups are the same as in Figs. 3 and 5.

The estimated crown age of the *A. sylvestris* complex, based on the plastid data (Fig. 7), was 1.72 Ma (95% HPD: 2.58–0.93 Ma), which is earlier than the estimate based on ITS data, but the 95% HPD intervals obtained for both datasets largely overlapped. The topology of the tree was also distinct: all plastid haplogroups were identified as clades (compare with Figs. 5 and S3). The *L* haplogroup was determined to be sister to the *F* + *E* clades. The divergence within the European group began approximately 0.44 Ma (95% HPD: 0.72–0.20 Ma).

### Fruit indumentum, geography and molecular phylogeny

A major morphological character used in the taxonomy of *Anthriscus sylvestris* s.l. is fruit indumentum. Fruits are devoid of hairs in *A. sylvestris* subsp. sylvestris and subsp. *alpina*, *A. nitida*, *A. schmalhausenii*, and *A. lamprocarpa*. Conversely, they are adorned with tuberculate bristles in *A. sylvestris* subsp. *nemorosa* and subsp. *fumarioides* (Fig. 1). This distinction displays a marked geographical pattern: tuberculate/bristled fruits are predominantly found in the Balkans, the southern region of the Apennine Peninsula, Asia Minor, and the Himalayas. They are sporadically encountered in eastern Asia and tropical East Africa along with the glabrous-fruited morphotype. Glabrous fruits are present in all accessions from North Africa as well as central and western Europe. This distribution pattern does not align with any of the molecular markers investigated. Notably, both glabrous and tuberculate-fruited specimens exist within the *E*, *A*, and *L* plastid haplogroups (e.g., Figs. 5 and 7). Similarly, the *Nit* and *Syl* ribotype groups each encompass both morphotypes. To illustrate, the representatives of tuberculate-fruited *A. s*. subsp. *nemorosa* from the Peloponnese possess the *Nit* ribotype, whereas their morphologically identical counterparts from southern Italy bear the *Syl* ribotype.

## Discussion

### Taxonomy versus molecular trees

While each molecular marker analyzed in this study displays specific biogeographical or ecological patterns, these patterns do not align with morphological markers such as leaf architecture and fruit indumentum. As a result, they also diverge from the current taxonomy of the genus.

Plastid data suggest a western Asian origin for *Anthriscus* and its sect. *Cacosciadium*. This agrees with previous results^10^ reconstructing Mediterranean or Irano-Turanian ancestral areas for most core apioid clades (or tribes). Interestingly, pollen microfossils that may be attributed to the euapioid clade (including subtribe Scandicinae that encompasses *Anthriscus*) were recovered from Oligo-Miocene sediments of eastern Anatolia^44^ thus supporting the aforementioned scenario. The split between Central Asian *Kozlovia* and western Eurasian *Anthriscus*, taken in this study as secondary calibration point, was estimated to have occurred in the upper Miocene, c. 9 Ma ^10^, an epoch characterized by a slowly drying climate that might have spurred vicariance events^3^. However, the major diversification trigger was most probably the onset of the Pleistocene glaciations. An exemplary pair of sister species originating during the Ice Age is *Anthriscus tenerrima* and *A. caucalis*. The former is restricted to the eastern Mediterranean (Greece and western Turkey), while the latter is now a common weed spreading due to its small fruits covered by tuberculate bristles^11^. In Spain and south-western France, *A. caucalis* is also represented by a glabrous-fruited variety. Interestingly, a similar polymorphism—the presence of plants with glabrous fruits and with tuberculate fruits within the same population—is characteristic of *A. tenerrima*.

Such polymorphism in annual umbellifers, often expressed within a single plant as heterocarpy, likely reflects alternative dispersal strategies: glabrous fruits are dispersed by gravity, facilitating local population renewal, while bristled fruits are dispersed on animal fur over greater distances^45^. The ancestor of *A. caucalis* and *A. tenerrima* might have been polymorphic with respect to fruit indumentum^12^. This polymorphism was inherited by the descendant species, which originated due to a vicariance event, surviving the Ice Age in two major refugia: the Pyrenean Peninsula and the Balkan Peninsula/Asia Minor. The bristled-fruited variety of *A. caucalis* spread with human activity and has become a noxious weed, while its glabrous-fruited variety remained in the refugial area.

Molecular data included in this study do not support the present taxonomic treatment of the sect. *Cacosciadium*, as all taxa, regardless of their rank, are nested within the geographically widespread *A. sylvestris* subsp. *sylvestris*. However, it is important to remember that gene trees do not always reflect species trees, particularly in cases of recent speciation with incomplete lineage sorting^46^. Peripatric and sympatric speciation events practically always result in one species being nested within another in a phylogenetic tree. Interspecific phylogenies, geographical ranges, and adaptive shifts are often used to infer geographical modes of speciation albeit there are some caveats of such inferences^46^. In the revision of *Anthriscus* based on morphological data only, *A. nemorosa* and *A. fumarioides* were reduced to the rank of subspecies of *A. sylvestris*, while *A. nitida*, *A. lamprocarpa*, and *A. schmalhausenii* were maintained as distinct species due to their morphological and ecological disparity with *A. sylvestris*^11,12^.

*Anthriscus nitida* is sympatric with *A. sylvestris* subsp. *sylvestris* throughout its range, but these two taxa significantly differ in habitat preferences. The former occurs in the mountainous beech-fir forest belt, while the latter inhabits rich and moist anthropogenic habitats such as road verges, meadows, and forest margins^11^. Substantial range overlap and adaptive shifts are often regarded as hallmarks of sympatric speciation, but such an interpretation may be overreaching because range overlap may be secondary while adaptive shifts often accompany ‘standard’ vicariant speciation^46^. Although lowland riparian forests were hypothesized to be the primary habitats of *A. sylvestris* subsp. *sylvestris*^11,12^, this taxon occurs almost exclusively in secondary anthropogenic habitats in Central Europe, hence its wide geographical range may be quite recent and related to human activities. In contrast, the range of *A. nitida* is similar to that of other species inhabiting mountainous beech-fir forests, particularly *Abies alba*^7^. It is possible that *A. nitida* originated in the late Pleistocene in one of the southern refugia of beech-fir forests, particularly in the Balkans. Its speciation would be, therefore, allopatric rather than sympatric. However, the delimitation of *A. nitida* is contentious as discussed later in this paper.

*Anthriscus lamprocarpa* differs from its parapatric cousin *A. sylvestris* subsp. *nemorosa* in having a biennial habit and straw-yellow glabrous fruits, in contrast to the perennial lifespan and brown/black tuberculate fruits of the latter^11^. However, two intermediate populations from southern Turkey have been identified and described as *A. lamprocarpa* subsp. *chelikii*^47^. This taxon might be of hybrid origin; alternatively, *A. sylvestris* subsp. *nemorosa*, *A. lamprocarpa* subsp. *cheliki*, and *A. lamprocarpa* subsp. *lamprocarpa* could represent clinal variation since fruit indumentum, color, and cuticle are under strong selection pressure, particularly related to fruit dispersal^48,49^ and avoidance of seed predation^50^. Hairy, bristled, or spiny versus glabrous fruits are known in many species of umbellifers and usually regarded as adaptations to dispersal on animal fur or by gravity^45,51^. However, the narrow, beaked mericarps of *A. sylvestris*, even without any specialized appendages, have quite good attachment potential to animal fur, for example of wild boar and sheep^52^.

Seed color may represent an adaptive response to fruit predation. In the legume species *Acmispon wrangelianus*, which is polymorphic with respect to seed color, the latter appears to match the soil color^53^. *Anthriscus lamprocarpa* inhabits warmer and dryer habitats than *A. sylvestris* subsp. *nemorosa,* so its straw-colored fruits may better camouflage among dried remnants of plants, whereas the brownish fruits of the latter may be less visible on more moist, brownish soil. Interestingly, *A. lamprocarpa* is morphologically similar to North African populations of *A. sylvestris* subsp. *sylvestris*, sometimes recognized as *A. sylvestris* subsp. *mollis* (Boiss. & Reut.) Maire, with the former suggested to descend from the latter through peripatric speciation^12^. However, our results indicate that *A. lamprocarpa* is of Anatolian rather than North African origin, while African ‘subsp. *mollis*’ may be its descendant rather than the ancestor.

*Anthriscus schmalhausenii* was initially described in the genus *Chaerophyllum* based on its sparsely divided leaves with broad lobes resembling those of *C. aromaticum*. Subsequently, it was reclassified into *Anthriscus* based on flower and fruit characteristics. Within *Anthriscus,* it was assigned to sect. *Caroides*, alongside other Caucasian species, *A. kotschyi* and *A. ruprechtii*^54^. In the most recent treatment of the genus^11,12^, its similarity to *A. nitida* in terms of leaf shape and division was noted but interpreted as a case of parallel evolution adapting to shady deciduous forests. Our research situates this taxon within the western and central Asiatic plastid clade (*A*) and within the *Syl* ribotype group (we failed to amplify *waxy* for this taxon). This species is found in Georgia and adjacent regions of Russia (Adygea, Krasnodar Krai), which constituted the Colchis Glacial Refugium – an important sanctuary for forest species.

In conclusion, there is no evidence supporting sympatric speciation in *Anthriscus sylvestris* s.l. Instead, geographical distance and isolation within glacial refugia appear to have played significant roles as drivers of local differentiation and speciation in this group.

### Migration routes around the Mediterranean

The circum-Mediterranean distribution of *Anthriscus sylvestris* complex raises questions about the migration routes and whether different migration waves have met closing the distribution ring.

Levantine *A. lamprocarpa* has been speculated to descend from the North African populations of *A. sylvestris*, the latter originating from the Iberian populations, which would imply that two migration waves met in southern Turkey^12^, along the Anatolian diagonal^55^. However, our analyses of plastid data allow to refute this hypothesis and suggest two alternative scenarios. In maximum likelihood plastid DNA tree, the European accessions (*E*) form a clade nested in North African polytomy (*F*), which is nested in Middle Eastern *L* grade. Such a topology suggests that the migration of the *A. sylvestris* complex from Middle East to Europe did not occur across the Turkish Straits, but around the Mediterranean: through northern Africa to the Iberian Peninsula and then eastwards to the Balkans, the area of possible secondary contact with the *L* lineage. The presence of tuberculate fruits in the Balkans and southern Italy—a character that is common in Asiatic populations while being absent in western Europe—suggests subsequent gene flow from Asia to the Balkans. We are not aware of any other plant taxon with such a dispersal pattern around the Mediterranean, although floristic links between SW Asia and NW Africa have already been postulated^56^. Moreover, such a scenario has interesting implications for the biogeography of the Mediterranean, because it entails the existence of a barrier between Asia Minor and the Balkans, whereas, throughout the Pleistocene, these areas were connected by land bridges^57^.

However, the biogeographic scenario suggesting migration around the Mediterranean does not receive strong support. According to BEAST analyses, the Levantine (*L*) and North African (*F*) groups form clades rather than grades. Such a topology indicates an alternative scenario involving a series of vicariance events, with a common ancestor initially widespread across Asia Minor, North Africa, and Europe. Subsequently, this ancestor became fragmented into three distinct groups—*L*, *F*, and *E*—due to the geographic barriers of the Turkish Straits and the Strait of Gibraltar. The western *L* + *F* + *E* group was isolated from the eastern group, which combined Western and Central Asia (*A*) and the Far Eastern (*C*) clades, by the Anatolian diagonal^58^.

### Reticulate evolution in the Anthriscus sylvestris complex

There are three primary sources of genetic variation that underlie adaptive ecological niche shifts: pre-existing diversity, the accumulation of new mutations, and the acquisition of beneficial alleles from other lineages. Studies that quantify the relative importance of these sources are scarce. While some studies point to one major source of variation, namely standing variation^59^, others demonstrate the input of all three sources^60^. It has been argued that the advantage of introgression or reticulate evolution is not merely the admixture of beneficial alleles but most of all the reassembly of old genetic variants into novel combinations^16^. This “combinatorial” mechanism might not only facilitate rapid speciation but also adaptive radiation and sympatric speciation, and it might contribute to variation in speciation rates among lineages. Among angiosperms, events of reticulation in the evolution and speciation of many taxa have been ascertained; however, the contribution of admixture variation has rarely been evaluated^60,61^. We hypothesize that this mechanism was involved in the ecological diversification of *Anthriscus sylvestris* complex in Europe.

The incongruence between plastid and nuclear markers, as well as morphological traits such as fruit indumentum, particularly in the Balkans and Central Europe, suggests significant gene flow of possibly adaptive character. The most striking discovery is that, based on ITS variation, Dalmatian mountainous *A. sylvestris* subsp. *fumarioides* and Balkan representatives of subsp. *nemorosa*, both having tuberculate fruits, are not related to the specimens of subsp. *nemorosa* from Turkey, but rather to the glabrous-fruited *A. nitida* from the Balkans and the Carpathians (ribotype *Nit*). Surprisingly, mountainous *A*. *sylvestris* subsp. *alpina* and *A. nitida* from France have ribotype *Syl*, which is characteristic of lowland *A. sylvestris* subsp. *sylvestris.* The border between these ribotypes seems to align with the border between the Carpathians and the Sudeten Mountains, with Carpathian specimens of *A. nitida* having type *Nit*, whereas *nitida*-like plants from the Sudeten Mountains have type *Syl*.

The difference in leaf form between *A. sylvestris* and *A. nitida* follows a well-established pattern: leaves of plants of shady and moist habitats tend to be broader and less divided than those of plants inhabiting dryer and more open habitats^62,63^. It is therefore possible that “true” *A. nitida* comprises only plants from the Carpathians and the Balkans, while *nitida*-like forms from the Sudeten and the Alps evolved from *A. sylvestris* with the influx of adaptive genes from *A. nitida*. This influx is also suggested by the distribution pattern of *waxy* alleles: representatives of western *A. nitida* and *A. sylvestris* subsp. *alpina* have *waxy* alleles closely related to those in eastern *A. nitida* and *A. sylvestris* subsp. *fumarioides* while maintaining the *Syl* ribotype of lowland *A. sylvestris* subsp. *sylvestris*.

Our data suggest that the ecological diversity in the *A. sylvestris* complex is relatively recent. The ancestor of the European lineage likely immigrated to Europe approximately 0.44 Mya, prior to the Elster glaciation. Its history in Europe may have been similar to that of other European taxa, involving successive retreats to glacial refugia, divergent evolution in these refugia, followed by re-colonization and secondary contacts among previously isolated populations. For example, intensive reticulate evolution in glacial refugia has been documented for European oaks ^64^, while the Alpine lake whitefish species complex likely arose from a hybrid swarm of at least two glacial refugial lineages^65^.

A comprehensive evaluation of the reticulate evolution hypothesis in the *A. sylvestris* complex requires genome-wide study, which is currently underway.

## Conclusions

The current study offers significant insights into the taxonomy, speciation, and biogeography of *Anthriscus sylvestris* complex. Contrary to the notion of sympatric speciation in Central Europe, our findings do not reject the hypothesis that geographical isolation within glacial refugia was the dominant driver for differentiation within this group.

Plastid data corroborate previous suggestions of a western Asiatic origin for *Anthriscus* and its section *Cacosciadium*. Within the latter, the plastid data challenge the present taxonomic treatment as plastid haplotypes align with the geographic distribution of the samples rather than their taxonomy. Plastid trees suggest two alternative biogeographic scenarios. The first, and better supported, implies a migration around the Mediterranean. The second postulates a series of vicariance events resulting from geographical barriers that arose during the Ice Age. Both scenarios suggest that the Balkans were the place of secondary contact between long-isolated lineages. In contrast, nuclear data appear to divide into lowland and montane alleles, but the support for this pattern is not particularly strong.

Considering the molecular, ecological, and biogeographic data, it is evident that the diversification of *A. sylvestris* s.l. is a complex interplay of geographical, climatic, and ecological factors, as well as shaped by gene flow between previously isolated lineages. Future studies may benefit from expanded molecular datasets, coupled with detailed ecological and morphological analyses, to provide a clearer understanding of the evolutionary history of this taxon.

## Supplementary Information

Supplementary Information.

## Data Availability

All sequences are deposited in GenBank: voucher data and accession numbers are given in Table S1. Sequence matrices and trees are deposited in the University of Warsaw repository danebadawcze.uw.edu.pl. DOI: 10.58132/WACYXJ.
