# Supplementary material for: Phylogeographic evidence reveals multiple colonization events and a secondary contact zone in the Balkans for the Anthriscus sylvestris complex (Apiaceae)
Source: Sci Rep. 2025 Aug 26;15:31497. doi: 10.1038/s41598-025-15409-7 (PMC12381290; doi:10.1038/s41598-025-15409-7)
Supplement: Supplementary file 1 — Supplementary Information. [file 41598_2025_15409_MOESM1_ESM.docx]

Phylogeographic evidence reveals multiple colonization events and a secondary contact zone in the Balkans for the *Anthriscus sylvestris* complex (Apiaceae)

## Renata Kurzyna-Młynik^1^, Łukasz Banasiak^1^, Marcin Piwczyński^2^, Paulina Trzeciak^3^, Mergi Daba Dinka^1^, Alexei A. Oskolski^4^ & Krzysztof Spalik^1*^

^1^Institute of Evolutionary Biology, Faculty of Biology, Biological and Chemical Research Centre, University of Warsaw, Warsaw, Poland. ^2^Department of Plant Physiology and Biotechnology, Nicolaus Copernicus University in Toruń, Toruń, Poland. ^3^Independent researcher, Toruń, Poland. ^4^Department of Botany and Plant Biotechnology, University of Johannesburg, Johannesburg, South Africa. *email: [k.spalik@uw.edu.pl](mailto:k.spalik@uw.edu.pl)

**Supplementary Table S1**. Representatives of *Anthriscus* and outgroups examined in this study with respective GenBank reference numbers, and ribo- and/or haplogroup status if applicable. Sequences obtained for previous studies are identified with an asterisk behind the reference number.

| Taxon name | ID | Voucher | Estimated coordinates (decimal), radius | ITS | *waxy* | *rpoB*-*trnC* | *trnS*-*trnG* | *psbA*-*trnH* | Ribo- and/or haplogroups |
| --- | --- | --- | --- | --- | --- | --- | --- | --- | --- |
| *Anthriscus caucalis* M.Bieb. | #0078 | Russia, Krasnodar Krai, right bank of Kuban river, 2 km above stanitsa (village) Starokorsunskaya, 19 km E from Kuban, 12 May 1995, *B.Korotyaev* (LE) | 45.07 39.33 4.300 km | ON180545 |  | ON584061 | ON637404 | ON637302 |  |
| *Anthriscus caucalis* M.Bieb. | #1041 | Spain, Granada, Sierra Nevada, along road from Granada to Veleta, Cueva de la Higuera, 1740 m, 16 April 1997, *S.L.Jury* *17292* (RNG) | 37.14 -3.49 0.600 km | ON180544 |  | ON584060 | ON637403 | ON637301 |  |
| *Anthriscus cerefolium* var. *trichocarpa* Neilr. | #1047 | Uzbekistan, Western Tian-Shan Mtns, Chotkal Range, Aksakata Valley, c 1100 m, 7 June 1989, *K.Spalik* *s.n.* (WA 0000071576/0000072522) | 41.36 69.92 2.100 km | DQ379384* |  | ON584059 | ON637402 | ON637300 |  |
| *Anthriscus cerefolium* var. *trichocarpa* Neilr. | #1380 | Armenia, Syunik prov., Meghri municipality, near Agarak, Zangezur Mountains, mt. Khamli, 3 April 1967, *Y.I.Mulkidjanian* (LE) | 38.86 46.17 3.300 km | ON180541 |  |  |  |  |  |
| *Anthriscus cerefolium* var. *trichocarpa* Neilr. | #1381 | Azerbaijan, Ganja (formerly Kirovabad), valley of Gəncəçay, 1948, *A.A.Grossheim, I.A.Ilinskaja & M.I.Kirpichnikov* (LE) | 40.70 46.40 1.993 km | ON180542 |  |  |  |  |  |
| *Anthriscus cerefolium* (L.) Hoffm. | #0080 | Bulgaria, Black Sea coast, N of Burgas, East Stara Planina, hill Bibernata, village Kableshkovo, 250 m, 26 May 1999, *Optima Iter IX* *674* (RNG) | 42.67 27.56 2.123 km | MK142888* | ON584007 | MK142856* | ON637401 | ON637299 |  |
| *Anthriscus cerefolium* (L.) Hoffm. | #1184 | Georgia, Samtskhe–Javakheti region, near Borjomi, 19 June 1903, *I.Borodin* *432* (LE) | 41.84 43.39 3.136 km | ON180543 |  |  |  |  |  |
| *Anthriscus cerefolium* (L.) Hoffm. | #1377 | Russia, Krasnodar Krai, near Maykop, 1921 1924, *N.L.Pastukhov* (LE) | 44.61 40.10 5.350 km | ON180538 |  |  |  |  |  |
| *Anthriscus cerefolium* (L.) Hoffm. | #1378 | Azerbaijan, Lənkəran, 27 April 1960, *G.F.Achundov* (LE) | 38.77 48.84 4.880 km | ON180539 |  |  |  |  |  |
| *Anthriscus cerefolium* (L.) Hoffm. | #1379 | Russia, Krasnodar Krai, near Maykop, 7 May 1911, *N.Shestunov* (LE) | 44.58 40.10 1.752 km | ON180540 |  |  |  |  |  |
| *Anthriscus kotschyi* Fenzl ex Boiss. & Balansa | #0081 | Turkey, Artvin prov., Şavşat distr., Şavşat range [= Karçkal mtns. ?], 1889, *I.Kurkov* (LE) | 41.37 42.04 40.000 km | ON180552 |  | ON584066 | ON637409 | ON637307 |  |
| *Anthriscus kotschyi* Fenzl ex Boiss. & Balansa | #1223 | Turkey, C5 Niğde Prov., Bolkar Dağları, path from Yalaklar to Madenköy, below pass 3094 m W of Köpüktaş, 3094 m, 31 August 1997, *K.Spalik & A.Żochowska* *s.n.* (WA 0000071586) | 37.44 34.60 0.900 km | ON180529 |  |  |  |  |  |
| *Anthriscus kotschyi* Fenzl ex Boiss. & Balansa | #1264 | Turkey, Aladağlar, Narpuz Deresi gorge, c 2750 m, 9 September 1997, *K.Spalik & A.Żochowska* *s.n.* (WA 0000071656) | 37.83 35.14 0.500 km | ON180554 | ON584006 | ON584067 | ON637410 | ON637308 |  |
| *Anthriscus kotschyi* Fenzl ex Boiss. & Balansa | #1383 | Georgia, Adjara, Shavsheti range, Mt. Chirukhi, 28 July 1951, *A.Dmitrieva* (LE) | 41.46 42.48 3.000 km | ON180553 |  |  |  |  |  |
| *Anthriscus lamprocarpa* Boiss. | #0086 | Israel, Carmel, Keren Carmel Reserve, near Muhraqa Monastery, 19 March 1993, *K.Spalik* *s.n.* (WA 0000071670) | 32.67 35.09 0.606 km | ON180562 |  | ON584068 | ON637411 | ON637309 | *Syl, L* |
| *Anthriscus lamprocarpa* Boiss. | #1052 | Jordan, Irbid gov., Umm Qais, 23 March 1995, *J.Lahham & A.El-Oqlah* *8* (ILL) | 32.65 35.68 1.488 km | ON180531 |  | ON584070 | ON637413 | ON637311 | *Syl, L* |
| *Anthriscus lamprocarpa* Boiss. | #1053 | Israel, Lower Galilee, Mount Tabor, 4 April 1993, *K.Spalik* *s.n.* (WA 0000071667) | 32.69 35.39 0.597 km | ON180563 |  |  |  |  | *Syl* |
| *Anthriscus lamprocarpa* Boiss. | #1266 | Syria, Mt. Hermon, Banias, at the source of the Jordan River, 3 April 1993, *K.Spalik* *s.n.* (WA 0000071661) | 33.24 35.68 0.682 km | ON180566 |  |  |  |  | *Syl* |
| *Anthriscus lamprocarpa* Boiss. | #1269 | Turkey, Mersin (formerly İçel) prov., Mut, Adras Dağı, Mut to Ermenek, 1300 m, 14 May 1965, *M.J.E.Coode & B.M.G.Jones* *970* (E E00183146) | 36.61 33.14 1.100 km | ON180564 | ON584044 | ON584071 | ON637414 | ON637312 | *Syl, Afroasiatic group, L* |
| *Anthriscus lamprocarpa* Boiss. | #1271 | Turkey, Adana prov., Bahçe distr., Dumanlı Dağ above Haruniye, 1200 m, 19 April 1957, *P.H.Davis & I.C.Hedge* *D26843* (E E00183144) | 37.27 36.51 0.926 km | ON180565 |  | ON584069 | ON637412 | ON637310 | *Syl, L* |
| *Anthriscus nitida* (Wahlenb.) Hazsl. | #0087 | Bulgaria, Rila Mountains, Musala, forests around Borovets, 42°20′N 23°45′E, 1250 m, 27 July 1985, *M.F.Gardner & S.G.Gardner* *3150* (E E00011024) | 42.27 23.61 4.500 km | ON180575 | ON584049 | ON584077 | ON637420 | ON637318 | *Nit, Euromontane group, E* |
| *Anthriscus nitida* (Wahlenb.) Hazsl. | #1057 | France, Haut-Rhin dept., Lautenbach, by Boenlesgrab, 865 m, 4 July 1982, *J.-P.Reduron* *1982070702* (WA 0000071703) | 47.94 7.16 2.182 km | ON180579 | ON584046 | ON584078 | ON637421 | ON637319 | *Syl, Euromontane group, E* |
| *Anthriscus nitida* (Wahlenb.) Hazsl. | #1058 | Poland, Leoncina (Tarnawka) forest by the San River near Przemyśl, 9 May 1990, *K.Spalik* *s.n.* (WA 0000029849) | 49.78 22.39 3.271 km | ON180576 |  |  |  |  | *Nit* |
| *Anthriscus nitida* (Wahlenb.) Hazsl. | #1059 | France, Haut-Rhin dept., Boelensgrab, 28 September 1991, *K.Spalik* *s.n.* (WA 0000071697) | 47.98 7.15 0.340 km | ON180581 |  |  |  |  | *Syl* |
| *Anthriscus nitida* (Wahlenb.) Hazsl. | #1185 | Romania, Transylvania, Braşov, by the Valea cu Apă River, 630 m, 3 June 1973, *M.Danciu* (BM) | 45.63 25.57 0.988 km | ON180573 |  |  |  |  | *Nit* |
| *Anthriscus nitida* (Wahlenb.) Hazsl. | #1259 | France, Haut-Rhin Dept., Boelensgrab near Lautenbach, 870 m, 4 September 1996, *J.-P.Reduron* *s.n.* (WA 0000071583) | 47.94 7.16 0.700 km | ON180580 | ON584048 | ON584079 | ON637422 | ON637320 | *Syl, Euromontane group, E* |
| *Anthriscus nitida* (Wahlenb.) Hazsl. | #1261 | Poland, Leoncina (Tarnawka) forest by the San River near Przemyśl, 9 May 1990, *K.Spalik* *s.n.* (WA 0000071707) | 49.78 22.39 3.271 km | ON180577 |  |  |  |  | *Nit* |
| *Anthriscus nitida* (Wahlenb.) Hazsl. | #1262 | France, Haut-Rhin dept., border between France and Switzerland, along the "Lucelle" stream, c 500 m, 29 September 1991, *K.Spalik & J.-P.Reduron* *s.n.* (WA 0000071584) | 47.44 7.31 5.200 km | ON180533 |  |  |  |  | *Syl* |
| *Anthriscus nitida* (Wahlenb.) Hazsl. | #1480 | Romania, Transylvania, Braşov, by the Valea cu Apă River, 631 m, 4 June 1973, *M.Danciu* *3467* (BM) | 45.63 25.57 0.988 km | ON180574 |  |  |  |  | *Nit* |
| *Anthriscus nitida* (Wahlenb.) Hazsl. | #1516 | Poland, Pieniny Mountains, Ociemny Wierch, 17 September 2007, *Ł.Banasiak* *s.n.* (WA 0000071567) | 49.42 20.43 0.352 km | ON180578 | ON584045 | ON584080 | ON637423 | ON637321 | *Nit, Euromontane group, E* |
| *Anthriscus nitida* (Wahlenb.) Hazsl. | #1751 | Poland, road from tourist parking at the W end of Zamkowa st. to Czorsztyn castle, 19 September 2007, *Ł.Banasiak* *s.n.* (WA 0000071570) | 49.44 20.32 2.538 km | ON064027 | ON584047 |  |  |  | *Int, Euromontane group* |
| *Anthriscus nitida* (Wahlenb.) Hazsl. | #2645 | Poland, 49.3129°N 22.4462°E, 548 m, 15 June 2021, *M.Piwczyński & P.Trzeciak* (WA 0000165468) |  | PP110820 |  |  |  |  | *Nit* |
| *Anthriscus nitida* (Wahlenb.) Hazsl. | #2647 | Poland, 49.2486°N 22.3974°E, 493 m, 15 June 2021, *M.Piwczyński & P.Trzeciak* (WA 0000165470) |  | PP110821 |  |  |  |  | *Nit* |
| *Anthriscus nitida* (Wahlenb.) Hazsl. | #2648 | Poland, 49.2265°N 22.3619°E, 532 m, 15 June 2021, *M.Piwczyński & P.Trzeciak* (WA 0000165471) |  | PP110822 |  |  |  |  | *Nit* |
| *Anthriscus nitida* (Wahlenb.) Hazsl. | #2649 | Poland, 49.1823°N 22.3998°E, 646 m, 15 June 2021, *M.Piwczyński & P.Trzeciak* (WA 0000165472) |  | PP110823 |  |  |  |  | *Nit* |
| *Anthriscus nitida* (Wahlenb.) Hazsl. | #2650 | Poland, 49.146°N 22.5183°E, 727 m, 15 June 2021, *M.Piwczyński & P.Trzeciak* (WA 0000165473) |  | PP110824 |  |  |  |  | *Nit* |
| *Anthriscus nitida* (Wahlenb.) Hazsl. | #2651 | Poland, 49.1661°N 22.5859°E, 664 m, 15 June 2021, *M.Piwczyński & P.Trzeciak* (WA 0000165474) |  | PP110825 |  |  |  |  | *Nit* |
| *Anthriscus nitida* (Wahlenb.) Hazsl. | #2654 | Poland, 49.4583°N 22.6435°E, 426 m, 16 June 2021, *M.Piwczyński & P.Trzeciak* (WA 0000165477) |  | PP110826 |  |  |  |  | *Nit* |
| *Anthriscus nitida* (Wahlenb.) Hazsl. | #2656 | Poland, 49.5487°N 22.6063°E, 555 m, 16 June 2021, *M.Piwczyński & P.Trzeciak* (WA 0000165479) |  | PP110827 |  |  |  |  | *Nit* |
| *Anthriscus nitida* (Wahlenb.) Hazsl. | #2659 | Poland, 49.602°N 22.6349°E, 484 m, 16 June 2021, *M.Piwczyński & P.Trzeciak* (WA 0000165482) |  | PP110828 |  |  |  |  | *Nit* |
| *Anthriscus nitida* (Wahlenb.) Hazsl. | #2663 | Poland, 49.6329°N 22.7142°E, 361 m, 16 June 2021, *M.Piwczyński & P.Trzeciak* (WA 0000165486) |  | PP110829 |  |  |  |  | *Nit* |
| *Anthriscus nitida* (Wahlenb.) Hazsl. | #2668 | Poland, 49.5044°N 21.1345°E, 422 m, 17 June 2021, *M.Piwczyński & P.Trzeciak* (WA 0000165491) |  | PP110831 |  |  |  |  | *Nit* |
| *Anthriscus nitida* (Wahlenb.) Hazsl. | #2669 | Poland, 49.4934°N 21.1467°E, 441 m, 17 June 2021, *M.Piwczyński & P.Trzeciak* (WA 0000165492) |  | PP110832 |  |  |  |  | *Nit* |
| *Anthriscus nitida* (Wahlenb.) Hazsl. | #2670 | Poland, 49.533°N 21.1039°E, 446 m, 17 June 2021, *M.Piwczyński & P.Trzeciak* (WA 0000165493) |  | PP110833 |  |  |  |  | *Nit* |
| *Anthriscus nitida* (Wahlenb.) Hazsl. | #2671 | Poland, 49.5412°N 21.0905°E, 439 m, 17 June 2021, *M.Piwczyński & P.Trzeciak* (WA 0000165494) |  | PP110834 |  |  |  |  | *Nit* |
| *Anthriscus nitida* (Wahlenb.) Hazsl. | #2672 | Poland, 50.243°N 16.7525°E, 752 m, 25 June 2021, *M.Piwczyński* (WA 0000165495) |  | PP110836 |  |  |  |  | *Syl* |
| *Anthriscus nitida* (Wahlenb.) Hazsl. | #2673 | Poland, 50.2496°N 16.7547°E, 595 m, 25 June 2021, *M.Piwczyński* (WA 0000165496) |  | PP110837 |  |  |  |  | *Syl* |
| *Anthriscus nitida* (Wahlenb.) Hazsl. | #2674 | Poland, 50.2497°N 16.7548°E, 595 m, 25 June 2021, *M.Piwczyński* (WA 0000165497) |  | PP110838 |  |  |  |  | *Syl* |
| *Anthriscus nitida* (Wahlenb.) Hazsl. | #2675 | Poland, 50.267°N 16.781°E, 716 m, 25 June 2021, *M.Piwczyński* (WA 0000165498) |  | PP110839 |  |  |  |  | *Syl* |
| *Anthriscus nitida* (Wahlenb.) Hazsl. | #2676 | Poland, 50.2637°N 16.8309°E, 719 m, 25 June 2021, *M.Piwczyński* (WA 0000165499) |  | PP110840 |  |  |  |  | *Syl* |
| *Anthriscus nitida* (Wahlenb.) Hazsl. | #2677 | Poland, 50.2539°N 16.8537°E, 670 m, 25 June 2021, *M.Piwczyński* (WA 0000165500) |  | PP110841 |  |  |  |  | *Syl* |
| *Anthriscus nitida* (Wahlenb.) Hazsl. | #2678 | Poland, 50.2314°N 16.8844°E, 675 m, 25 June 2021, *M.Piwczyński* (WA 0000165501) |  | PP110842 |  |  |  |  | *Syl* |
| *Anthriscus nitida* (Wahlenb.) Hazsl. | #2680 | Poland, 50.4936°N 16.3654°E, 453 m, 26 June 2021, *M.Piwczyński* (WA 0000165503) |  | PP110843 |  |  |  |  | *Syl* |
| *Anthriscus nitida* (Wahlenb.) Hazsl. | #2682 | Poland, 50.4787°N 16.39°E, 638 m, 26 June 2021, *M.Piwczyński* (WA 0000165505) |  | PP110844 |  |  |  |  | *Syl* |
| *Anthriscus nitida* (Wahlenb.) Hazsl. | #2683 | Poland, 50.4522°N 16.3434°E, 777 m, 26 June 2021, *M.Piwczyński* (WA 0000165506) |  | PP110845 |  |  |  |  | *Syl* |
| *Anthriscus nitida* (Wahlenb.) Hazsl. | #2686 | Poland, 49.4165°N 20.2203°E, 726 m, 21 July 2021, *M.Piwczyński* (WA 0000165509) |  | PP110947 |  |  |  |  | *Syl* |
| *Anthriscus nitida* (Wahlenb.) Hazsl. | #2687 | Poland, 49.4183°N 20.2273°E, 861 m, 21 July 2021, *M.Piwczyński* (WA 0000165510) |  | PP110948 |  |  |  |  | *Syl* |
| *Anthriscus nitida* (Wahlenb.) Hazsl. | #2688 | Poland, 49.4175°N 20.2488°E, 772 m, 21 July 2021, *M.Piwczyński* (WA 0000165511) |  | PP110949 |  |  |  |  | *Nit* |
| *Anthriscus nitida* (Wahlenb.) Hazsl. | #2689 | Poland, 49.4115°N 20.2501°E, 731 m, 21 July 2021, *M.Piwczyński* (WA 0000165512) |  | PP110950 |  |  |  |  | *Nit* |
| *Anthriscus nitida* (Wahlenb.) Hazsl. | #2691 | Poland, 49.4992°N 21.5859°E, 420 m, 17 June 2021, *M.Piwczyński & P.Trzeciak* (WA 0000165514) |  | PP110830 |  |  |  |  | *Nit* |
| *Anthriscus ruprechtii* Boiss. | #0082 | Georgia, Racha-Lechkhumi and Kvemo Svaneti region, sources of the Janauli River at the base of Jvari, 1900-2100 m, 8 August 1980, *R.Gagnidze, M.Tsiklauri, M.Mukbaniani & Sh.Shetekauri* (LE) | 42.69 42.55 0.550 km | ON180549 | ON584005 | ON584064 | ON637407 | ON637305 |  |
| *Anthriscus ruprechtii* Boiss. | #1367 | Russia, Kabardino-Balkaria, Chereksky distr., Bezengi rural loc., right bank of Cherek-Bezengiysky (Khulamsky), valley of river Suuk-Auz-Su, 2000-2500, 15 August 1988, *J.Menitsky, T.Popova & V.Nikolaev* *35* (LE) | 43.11 43.14 15.000 km | ON180548 |  |  |  |  |  |
| *Anthriscus ruprechtii* Boiss. | #1372 | Russia, Ingushetia, Nazranovsky distr., upper course of Armkhi river, 2500 m, 27 June 1971, *V.Prima* *s.n.* (LE) | 42.66 44.80 2.750 km | ON180550 |  |  |  |  |  |
| *Anthriscus ruprechtii* Boiss. | #1373 | Azerbaijan, Quba distr., upper course of Vəlvələçay (Babaçay) river, 5-6 km N of the summit of Babadağ, 3350 m, 20 July 1971, *V.Prima* *s.n.* (LE) | 41.07 48.30 6.623 km | ON180551 |  | ON584065 | ON637408 | ON637306 |  |
| *Anthriscus schmalhausenii* (Albov) Koso-Pol. | #0088 | Georgia, Abkhazia, Gulripshi distr., near Amtkel village, valley of Kholodnaja river, 6 km NNE from Tsebelda, 25 June 1989, *A.P.Dolmatova, V.I.Dorofeev, D.V.Geltman, L.I.Krupkina, A.K.Sytin & M.M.Ivanova* *2827* (LE) | 43.04 41.33 0.600 km | ON180558 |  | ON584094 | ON637437 | ON637335 | *Syl, A* |
| *Anthriscus schmalhausenii* (Albov) Koso-Pol. | #1376 | Russia, Krasnodar Krai, W Caucasus, Caucasian State Nature Reserve, Lago-Naki, Zhita, 21 July 1929, *A.I.Leskov & A.P.Rusalev* *468* (LE) | 44.05 40.00 8.783 km | ON180557 |  | ON584092 | ON637435 | ON637333 | *Syl, A* |
| *Anthriscus schmalhausenii* (Albov) Koso-Pol. | #1388 | Georgia, South Ossetia, Shuatzkhuri, swamp below Upper Shuatzkhuri, 1550 m, 27 July 1930, *E.Busch & N.Busch* (LE) | 42.23 43.97 52.307 km | ON180559 |  |  |  |  | *Syl* |
| *Anthriscus schmalhausenii* (Albov) Koso-Pol. | #1389 | Georgia, Imereti, Sokhumi, by the bridge over the Basla river ("Venetian Bridge"), 10 May 1902, *T.Alexeenko* *19065* | 43.02 41.05 2.614 km | ON180560 |  |  |  |  | *Syl* |
| *Anthriscus schmalhausenii* (Albov) Koso-Pol. | #2313 | Russia, Karachay-Cherkessia, basin of the Urup river, upper course of the Vlasenchikha river, near Bakhmut, 9 August 1945, *V.Grubov & M.Shin* (LE) | 43.87 41.06 1.254 km | ON180561 |  | ON584093 | ON637436 | ON637334 | *Syl, A* |
| *Anthriscus sylvestris x nitida* | #2646 | Poland, 49.2885°N 22.4164°E, 447375854 m, 15 June 2021, *M.Piwczyński & P.Trzeciak* (WA 0000165469) |  | PP110954 |  |  |  |  | *Nit* |
| *Anthriscus sylvestris x nitida* | #2653 | Poland, 49.4571°N 22.6438°E, 420939819 m, 16 June 2021, *M.Piwczyński & P.Trzeciak* (WA 0000165476) |  | PP110941 |  |  |  |  | *Nit* |
| *Anthriscus sylvestris x nitida* | #2657 | Poland, 49.5486°N 22.6063°E, 557205566 m, 16 June 2021, *M.Piwczyński & P.Trzeciak* (WA 0000165480) |  | PP110942 |  |  |  |  | *Syl* |
| *Anthriscus sylvestris x nitida* | #2665 | Poland, 49.466°N 21.6226°E, 460 m, 17 June 2021, *M.Piwczyński & P.Trzeciak* (WA 0000165488) |  | PP110943 |  |  |  |  | *Syl* |
| *Anthriscus sylvestris x nitida* | #2666 | Poland, 49.4984°N 21.4218°E, 422 m, 17 June 2021, *M.Piwczyński & P.Trzeciak* (WA 0000165489) |  | PP110944 |  |  |  |  | *Nit* |
| *Anthriscus sylvestris x nitida* | #2681 | Poland, 50.4847°N 16.3787°E, 571 m, 26 June 2021, *M.Piwczyński* (WA 0000165504) |  | PP110945 |  |  |  |  | *Syl* |
| *Anthriscus sylvestris x nitida* | #2684 | Poland, 50.5532°N 16.71°E, 380 m, 27 June 2021, *M.Piwczyński* (WA 0000165507) |  | PP110946 |  |  |  |  | *Syl* |
| *Anthriscus sylvestris x nitida* | #2690 | Poland, 49.4069°N 20.2086°E, 771 m, 21 July 2021, *M.Piwczyński* (WA 0000165513) |  | PP110951 |  |  |  |  | *Nit* |
| *Anthriscus sylvestris* subsp. *alpina* (Vill.) Gremli | #1063 | France, Isère dept., Chartreuse Mountains, below Bovinant pass, Oct 1991, *K.Spalik* *s.n.* (WA 0000071711) | 45.38 5.81 0.534 km | ON180530 | ON584050, ON584053 | ON584081 | ON637424 | ON637322 | *Syl, Euromontane group, E* |
| *Anthriscus sylvestris* subsp. *alpina* (Vill.) Gremli | #1064 | France, Pyrénées-Orientales dept., Cerdagne, gorges of Llo, 1500 m, *F.Sennen* *4415* (RNG) | 42.45 2.07 0.410 km | ON180556 |  | ON584083 | ON637426 | ON637324 | *Syl, E* |
| *Anthriscus sylvestris* subsp. *alpina* (Vill.) Gremli | #1186 | Switzerland, Jura Bernensis, Porrentruy distr., Bressaucourt, Jun 1894/Jun 1897, *K.G.Bernoulli* *3416* (BM) | 47.39 7.03 0.845 km | ON180555 |  | ON584082 | ON637425 | ON637323 | *Syl, E* |
| *Anthriscus sylvestris* subsp. *fumarioides* (Waldst. & Kit.) Spalik | #0085 | Bosnia and Herzegovina, Kajabaša near Travnik, 1100 m, Jun 1896, *E.Brandis* *s.n.* (E E00040942) | 44.25 17.65 0.581 km | ON180570 | ON584055 | ON584075 | ON637418 | ON637316 | *Nit, Euromontane group, E* |
| *Anthriscus sylvestris* subsp. *fumarioides* (Waldst. & Kit.) Spalik | #1188 | Slovenia, Inner Carniola, Nanos Plateau, near Razdrto [Präwald], 11 March 1913, *A.Paulin* *951* (BM) | 45.78 14.05 1.392 km | ON180567 |  | ON584074 | ON637417 | ON637315 | *Nit, E* |
| *Anthriscus sylvestris* subsp. *fumarioides* (Waldst. & Kit.) Spalik | #1189 | Slovenia, Primorje, Mt. Slavnik, 800-1000 m, Jun 1910, *C.Marchesetti* *1712* (BM BM000537128) | 45.53 13.98 0.910 km | ON180569 |  |  |  |  | *Nit* |
| *Anthriscus sylvestris* subsp. *fumarioides* (Waldst. & Kit.) Spalik | #1226 | Bosnia and Herzegovina, near Derventa, May 1880, *I.Dörfler* (E E00040941) | 44.98 17.91 1.523 km | ON180532 |  |  |  |  | *Nit* |
| *Anthriscus sylvestris* subsp. *fumarioides* (Waldst. & Kit.) Spalik | #1274 | Croatia, Dalmatia, Biokovo mountains, above Makarska, 43d 20m N 16d 55m E, 2 August 1984, *M.F.Gardner & S.G.Gardner* *2525* (E E00011026) | 43.31 17.06 1.418 km | ON180568 | ON584051, ON584052 | ON584073 | ON637416 | ON637314 | *Nit, Euromontane group, E* |
| *Anthriscus sylvestris* subsp. *nemorosa* (M.Bieb.) Koso-Pol. | #0084 | Russia, Karachay-Cherkessia, the upper Kuban River, near Khurzuk village, left bank of the Uzunkol river, ascent to the Krugozor Myrdy, 6 August 1989, *J.Menitsky, T.Popova, S.Kuzmenkova & V.Bjalt* *107* (LE) | 43.29 42.16 1.149 km | ON180603 | ON584036 | ON584107 | ON637450 | ON637348 | *Syl, Afroasiatic group, A* |
| *Anthriscus sylvestris* subsp. *nemorosa* (M.Bieb.) Koso-Pol. | #1067 | Russia, Dagestan, Tlyaratinsky distr., 3 km S from Tlyarata, 22 August 1989, *J.Menitsky, A.J.Magulaev, S.Kuzmenkova, V.Bjalt & T.D.Vyshenskaja* *125* (LE) | 42.07 46.35 1.020 km | ON180583 |  | ON584096 | ON637439 | ON637337 | *Syl, A* |
| *Anthriscus sylvestris* subsp. *nemorosa* (M.Bieb.) Koso-Pol. | #1068 | Turkey, Rize prov., Kaçkar Dağları, Kaçkar-Kavron group, c 2400 m, 15 August 1996, *K.Spalik* *s.n.* (WA 0000071579) | 40.88 41.14 0.550 km | ON180614 |  |  |  |  | *Syl* |
| *Anthriscus sylvestris* subsp. *nemorosa* (M.Bieb.) Koso-Pol. | #1070 | Italy, Calabria, Cosenza prov., Sila Grande c. 13 km ENE of Camigliatello Silano, Macchialonga, 1550 m, 11 June 1997, *Optima Iter VIII* *982* (RNG) | 39.37 16.60 1.419 km | ON180585 |  | ON584087 | ON637430 | ON637328 | *Syl, E* |
| *Anthriscus sylvestris* subsp. *nemorosa* (M.Bieb.) Koso-Pol. | #1071 | Italy, Calabria, Cosenza prov., Sila Grande, c. 6 km ENE of Camigliatello Silano; southern end of Cecita Lake, 1140 m, 13 June 1997, *Optima Iter VIII* *1456* (RNG) | 39.37 16.51 0.989 km | ON180586 | ON584009, ON584010 | ON584085 | ON637428 | ON637326 | *Syl, European lowland group, E* |
| *Anthriscus sylvestris* subsp. *nemorosa* (M.Bieb.) Koso-Pol. | #1072 | Italy, Calabria, Reggio di Calabria prov., Aspromonte, near the Sanctuary of Santa Maria di Polsi, Jun 1898, *G.Rigo* (RNG) | 38.16 15.96 0.850 km | ON180587 |  | ON584076 | ON637419 | ON637317 | *Syl, E* |
| *Anthriscus sylvestris* subsp. *nemorosa* (M.Bieb.) Koso-Pol. | #1192 | Georgia, Dusheti municipality, upper course of the Arghuni River, near Shatili village, near Georguninda, 28 July 1985, *J.Menitsky* (LE) | 42.66 45.16 3.136 km | ON180604 | ON584032 | ON584108 | ON637451 | ON637349 | *Syl, Afroasiatic group, A* |
| *Anthriscus sylvestris* subsp. *nemorosa* (M.Bieb.) Koso-Pol. | #1193 | Armenia, Kotayk prov., S slopes of Pambak mountains range, near Hankavan, 2400 m, 7 August 1986, *J.Menitsky, T.Popova, V.Nikitin & N.Medvedeva* (LE) | 40.65 44.49 0.900 km | ON180605 |  | ON584109 | ON637452 | ON637350 | *Syl, A* |
| *Anthriscus sylvestris* subsp. *nemorosa* (M.Bieb.) Koso-Pol. | #1194 | Azerbaijan, Talysh Mountains, Lerik dist., near Orand village, 29 June 1970, *J.Menitsky* (LE) | 38.75 48.34 1.480 km | ON180607 |  | ON584110 | ON637453 | ON637351 | *Syl, A* |
| *Anthriscus sylvestris* subsp. *nemorosa* (M.Bieb.) Koso-Pol. | #1195 | Russia, Dagestan, Akushinsky distr., forest Gumraduz between Akusha and Mugi, 1200-1400, 14 July 1898, *T.Alexeenko* *8612* (LE) | 42.29 47.38 2.860 km | ON180609 |  |  |  |  | *Syl* |
| *Anthriscus sylvestris* subsp. *nemorosa* (M.Bieb.) Koso-Pol. | #1196 | Armenia, Gegharkunik prov., N shore of lake Sevan, near Sevan (formerly Elenovka) town, 27 June 1927, *A.Schelkovnikov & E.Kara-Murza* (LE) | 40.56 44.95 0.819 km | ON180610 |  |  |  |  | *Syl* |
| *Anthriscus sylvestris* subsp. *nemorosa* (M.Bieb.) Koso-Pol. | #1197 | Armenia, Gegharkunik prov., N shore of lake Sevan, near Sevan (formerly Elenovka) town, 11 July 1960, *N.Savitch* (LE) | 40.56 44.95 0.819 km | ON180611 |  |  |  |  | *Syl* |
| *Anthriscus sylvestris* subsp. *nemorosa* (M.Bieb.) Koso-Pol. | #1198 | Armenia, Shirak prov., near Ghazanchi (formerly Kaikuli), 11 June 1934, *A.Takhtajan* (LE) | 41.07 43.84 1.626 km | ON180612 |  |  |  |  | *Syl* |
| *Anthriscus sylvestris* subsp. *nemorosa* (M.Bieb.) Koso-Pol. | #1217 | Bulgaria, Rila Mountains, near Suhoto lake, 1885 m, 7 July 2004, *C.Aedo et al.* *10418* (MA) | 42.06 23.57 0.768 km | ON180584 | ON584011 | ON584084 | ON637427 | ON637325 | *Syl, European lowland group, E* |
| *Anthriscus sylvestris* subsp. *nemorosa* (M.Bieb.) Koso-Pol. | #1267 | Turkey, Artvin prov., Çoruh, Kaçkar Dağları, Didvake massif, 2500 m, 8 August 1996, *K.Spalik* *s.n.* (WA 0000071675) | 40.99 41.29 0.258 km | ON180534 | ON584040 | ON584112 | ON637455 | ON637353 | *Syl, Afroasiatic group, A* |
| *Anthriscus sylvestris* subsp. *nemorosa* (M.Bieb.) Koso-Pol. | #1276 | Turkey, Aydın prov., Samsun Dağı above Güzelçamlı, 500-600 m, 24 April 1965, *P.H.Davis* *41687* (E E00183165) | 37.69 27.24 0.560 km | ON180591 |  | ON584072 | ON637415 | ON637313 | *Syl, L* |
| *Anthriscus sylvestris* subsp. *nemorosa* (M.Bieb.) Koso-Pol. | #1281 | Iran, Azerbaijan, Kuh-e Sahand, between Liqvan and Isperekhan, 2 July 1978, *M.Assadi & V.Mozaffarian* *30608* (E E00183160) | 37.83 46.42 1.476 km | ON180589 | ON584038, ON584039 | ON584098 | ON637441 | ON637339 | *Syl, Afroasiatic group, A* |
| *Anthriscus sylvestris* subsp. *nemorosa* (M.Bieb.) Koso-Pol. | #1283 | India, Himachal Pradesh state, Kullu distr., Parvati valley, Swajni maidan, 9500 ft, 25 May 1934, *C.E.Parkinson* *3949* (E E00183155) | 32.00 77.44 5.000 km | ON180592 |  | ON584099 | ON637442 | ON637340 | *Syl, A* |
| *Anthriscus sylvestris* subsp. *nemorosa* (M.Bieb.) Koso-Pol. | #1286 | Turkey, Ağrı prov., Doğubeyazıt distr., Küçük Ağrı Dağı (Little Ararat), near Serdar Bulak, 2300 m, 20 July 1966, *P.H.Davis* *46907* (E E00183150) | 39.68 44.41 1.032 km | ON180593 | ON584035 | ON584102 | ON637445 | ON637343 | *Syl, Afroasiatic group, A* |
| *Anthriscus sylvestris* subsp. *nemorosa* (M.Bieb.) Koso-Pol. | #1287 | Turkey, Hakkari prov., 10 km from Yüksekova to Şemdinli, 1950 m, 15 June 1966, *P.H.Davis* *45147* (E E00040978) | 37.52 44.38 0.744 km | ON180594 |  | ON584103 | ON637446 | ON637344 | *Syl, A* |
| *Anthriscus sylvestris* subsp. *nemorosa* (M.Bieb.) Koso-Pol. | #1289 | Turkey, Erzurum/Bayburt prov., Aşkale to Bayburt, Kop Dağı pass, 4 August 1965, *J.Lamond* *2601* (E E00183148) | 40.04 40.48 0.730 km | ON180595 |  | ON584104 | ON637447 | ON637345 | *Syl, A* |
| *Anthriscus sylvestris* subsp. *nemorosa* (M.Bieb.) Koso-Pol. | #1290 | Turkey, Kars prov., 4 km from Sarıkamış to Karaurgan, 2200 m, 15 July 1966, *P.H.Davis* *46610* (E E00183147) | 40.31 42.52 0.729 km | ON180596 |  | ON584105 | ON637448 | ON637346 | *Syl, A* |
| *Anthriscus sylvestris* subsp. *nemorosa* (M.Bieb.) Koso-Pol. | #1291 | Turkey, Bolu prov., Köroğlu Dağları, Aladağ, Kartalkaya, 2100-2200 m, 12 July 1962, *P.H.Davis & M.J.E.Coode* *37386* (E E00040937) | 40.57 31.81 1.632 km | ON180597 |  | ON584091 | ON637434 | ON637332 | *Syl, L* |
| *Anthriscus sylvestris* subsp. *nemorosa* (M.Bieb.) Koso-Pol. | #1293 | Italy, Sicily, Palermo, 35 km S from Cefalù, S from Isnello, 26 May 1979, *D.Davis & S.Sutton* *63816* (E E00040939) | 37.72 14.03 2.400 km | ON180598 |  |  |  |  | *Syl* |
| *Anthriscus sylvestris* subsp. *nemorosa* (M.Bieb.) Koso-Pol. | #1294 | Italy, Sicily, Messina, 15 km S from San Fratello, 31 May 1979, *D.Davis & S.Sutton* *64120* (E E00040940) | 37.91 14.60 1.522 km | ON180669 |  | ON584154 | ON637498 | ON637396 | *Syl, E* |
| *Anthriscus sylvestris* subsp. *nemorosa* (M.Bieb.) Koso-Pol. | #1295 | Nepal, Ghurchi Lagna, 14 May 1952, *O.Polunin, W.R.Sykes & L.H.J.Williams* *4076* (E E00040943) | 29.45 82.13 0.985 km | ON180599 | ON584030, ON584031 | ON584100 | ON637443 | ON637341 | *Syl, Intermediate, A* |
| *Anthriscus sylvestris* subsp. *nemorosa* (M.Bieb.) Koso-Pol. | #1296 | Italy, Sicily, Catania, about 10 km NW from Randazzo, Bosco di Cannata, 800-900 m, 3 June 1979, *D.Davis & S.Sutton* *64402* (E E00183140) | 37.95 15.02 1.331 km | ON180600 | ON584012 | ON584088 | ON637431 | ON637329 | *Syl, European lowland group, E* |
| *Anthriscus sylvestris* subsp. *nemorosa* (M.Bieb.) Koso-Pol. | #1299 | Iran, Semnan Prov., Shahrud County, Kuh-e Ghatri summit, 2550 m, 7 June 1973, *H.Foroughi* *9811* (E E00183134) | 36.76 55.03 0.600 km | ON180582 |  | ON584095 | ON637438 | ON637336 | *Syl, A* |
| *Anthriscus sylvestris* subsp. *nemorosa* (M.Bieb.) Koso-Pol. | #1301 | Iran, Central Alborz mountains, N of water shed above Ilika, 7500 ft, 24 June 1962, *P.Furse* *2795* (E E00183132) | 36.28 51.43 1.122 km | ON180601 | ON584034 | ON584106 | ON637449 | ON637347 | *Syl, Afroasiatic group, A* |
| *Anthriscus sylvestris* subsp. *nemorosa* (M.Bieb.) Koso-Pol. | #1333 | Russia, Dagestan, Rutulsky distr., above Tsakhur along river Samur, left bank, 1700-2000, 23 July 1978, *Z.Klotchkova, J.Menitsky & T.Popova* *553* (LE) | 41.66 47.14 1.355 km | ON180606 |  |  |  |  | *Syl* |
| *Anthriscus sylvestris* subsp. *nemorosa* (M.Bieb.) Koso-Pol. | #1385 | Russia, Karachay-Cherkessia, Teberda, 1938, *V.L.Komarov & N.V.Komarova* *194* (LE) | 43.45 41.74 4.450 km | ON180608 |  | ON584111 | ON637454 | ON637352 | *Syl, A* |
| *Anthriscus sylvestris* subsp. *nemorosa* (M.Bieb.) Koso-Pol. | #1387 | Azerbaijan, Qəbələ distr., left bank of Bumçay 4 km above Qəmərvan, 12 August 1965, *A.Shreter & M.Pimenov* *1029* (LE) | 41.09 47.81 0.803 km | ON180613 |  |  |  |  | *Syl* |
| *Anthriscus sylvestris* subsp. *nemorosa* (M.Bieb.) Koso-Pol. | #1475 | Montenegro, 14 July 1898, *A.Baldacci* (BM) | 42.70 19.40 110.000 km | ON180571 | ON584054 | ON584089 | ON637432 | ON637330 | *Nit, Euromontane group, E* |
| *Anthriscus sylvestris* subsp. *nemorosa* (M.Bieb.) Koso-Pol. | #1476 | Greece, Macedonia, Pisoderion, 1463 m, 23 June 1932, *A.H.G.Alston & N.Y.Sandwith* *438* (BM BM000998982) | 40.79 21.25 0.378 km | ON180572 |  | ON584090 | ON637433 | ON637331 | *Nit, E* |
| *Anthriscus sylvestris* subsp. *nemorosa* (M.Bieb.) Koso-Pol. | #1500 | Pakistan, Punjab, 28 May 1898, *J.F.Duthie* *21084* (BM BM000537148) | 31.00 72.00 375.000 km | ON180602 |  | ON584101 | ON637444 | ON637342 | *Syl, A* |
| *Anthriscus sylvestris* subsp. *nemorosa* (M.Bieb.) Koso-Pol. | #1508 | Croatia, 1877, *Shuttleworth* (BM) | 44.47 16.47 300.000 km | ON180588 |  | ON584097 | ON637440 | ON637338 | *Syl, A* |
| *Anthriscus sylvestris* subsp. *nemorosa* (M.Bieb.) Koso-Pol. | #1509 | Albania, Gjirokastër region, Mal Çajup [between Lunxhëri and Nemërçka Mountains], 4000 ft, 13 June 1933, *A.H.G.Alston & N.Y.Sandwith* *1671* (BM BM000537136) | 40.20 20.18 3.582 km | ON180590 |  | ON584086 | ON637429 | ON637327 | *Syl, E* |
| *Anthriscus sylvestris* subsp. *sylvestris* (L.) Hoffm. | #0083 | France, Haut-Rhin dept., Mulhouse, Forêt du Tannenwald, 8 September 1973, *J.-P.Reduron* *1973090801* (WA 0000071573) | 47.69 7.15 0.171 km | KT347715* | ON584019 | KT347743* | ON637495 | ON637393 | *Syl, European lowland group, E* |
| *Anthriscus sylvestris* subsp. *sylvestris* (L.) Hoffm. | #1073 | France, Isère dept., in the mountains of Annoisin, 17 May 1987, *G.Dutartre* *s.n.* (WA 0000071580) | 45.76 5.29 0.500 km | ON180661 |  |  |  |  | *Syl* |
| *Anthriscus sylvestris* subsp. *sylvestris* (L.) Hoffm. | #1074 | France, Nièvre dept., Cosne-Cours-sur-Loire, Sajots near Villechaud, 31 May 1987, *G.Dutartre* *s.n.* (WA 0000071581) | 47.41 2.93 3.591 km | ON180662 |  |  |  |  | *Syl* |
| *Anthriscus sylvestris* subsp. *sylvestris* (L.) Hoffm. | #1075 | Korea, 15 October 1965 (WA 0000071582) | 38.00 127.00 600.000 km | ON180650 |  | ON584122 | ON637465 | ON637363 | *Syl, C* |
| *Anthriscus sylvestris* subsp. *sylvestris* (L.) Hoffm. | #1078 | Spain, Adalusia, Granada, Barranco de San Jerónimo, 9 July 1971, *Casas* (MA) | 37.21 -3.58 2.600 km | ON180634 |  | ON584136 | ON637479 | ON637377 | *Syl, F* |
| *Anthriscus sylvestris* subsp. *sylvestris* (L.) Hoffm. | #1081 | France, Pyrénées-Orientales Dept., Cerdagne, gorges of Llo, 1500 m, 15 July 1919, *F.Sennen* *3693* (RNG) | 42.45 2.07 0.410 km | ON064024 | ON584020 |  |  |  | *Syl, European lowland group* |
| *Anthriscus sylvestris* subsp. *sylvestris* (L.) Hoffm. | #1082 | Algeria, Tell Atlas, Djurdjura range, near Tikjda, 1500 m, 3 June 1971, *P.H.Davis* *53083* (RNG) | 36.45 4.14 1.238 km | ON180635 | ON584026, ON584028 | ON584131 | ON637474 | ON637372 | *Syl, North African group, F* |
| *Anthriscus sylvestris* subsp. *sylvestris* (L.) Hoffm. | #1083 | United Kingdom, England, Surrrey, Woldingham, near South Hawke, 700 ft, 8 June 1975, *P.F.Cannon* (RNG) | 51.27 -0.03 0.389 km | ON180629 | ON584021 | ON584145 | ON637488 | ON637386 | *Syl, European lowland group, E* |
| *Anthriscus sylvestris* subsp. *sylvestris* (L.) Hoffm. | #1093 | Italy, Calabria, Reggio di Calabria prov., Aspromote, c. 10 km ESE of Gambarie, road to Canovai, 1460 m, 3 June 1997, *Optima Iter VIII* *227* (RNG) | 38.13 15.94 0.486 km | ON180643 |  | ON584152 | ON637496 | ON637394 | *Syl, E* |
| *Anthriscus sylvestris* subsp. *sylvestris* (L.) Hoffm. | #1094 | Italy, Sicily, Messina, 15 km S from San Fratello, 1000 m, 31 May 1979, *D.Davis & S.Sutton* *64120* (RNG) | 37.91 14.60 1.522 km | ON180668 | ON584022 | ON584138 | ON637481 | ON637379 | *Syl, European lowland group, E* |
| *Anthriscus sylvestris* subsp. *sylvestris* (L.) Hoffm. | #1095 | Russia, Moscow oblast, village Kolychevo, by Pakhra river, 14 June 1973, *E.E.Gogina & S.A.Thumanian* *163* (RNG) | 55.49 37.86 0.592 km | ON180644 |  | ON584153 | ON637497 | ON637395 | *Syl, E* |
| *Anthriscus sylvestris* subsp. *sylvestris* (L.) Hoffm. | #1096 | Denmark, Greater Copenhagen, near Sorgenfri station, 12 June 1970, *J.Svendsen* *259* (RNG) | 55.78 12.48 0.618 km | ON180646 |  | ON584137 | ON637480 | ON637378 | *Syl, E* |
| *Anthriscus sylvestris* subsp. *sylvestris* (L.) Hoffm. | #1097 | Iceland, Austur-Húnavatnssýsla, Blönduós, 30 July 1988, *P.M.D.Etherington* *88112* (RNG) | 65.66 -20.29 0.952 km | ON180626 | ON584041 | ON584144 | ON637487 | ON637385 | *Syl, Afroasiatic group, E* |
| *Anthriscus sylvestris* subsp. *sylvestris* (L.) Hoffm. | #1098 | Poland, Lesser Poland, Wadowice, 14 May 1970, *A.Jasiewicz & M.Sychowa* *248* (RNG) | 49.88 19.49 2.800 km | ON180641 |  | ON584150 | ON637493 | ON637391 | *Syl, E* |
| *Anthriscus sylvestris* subsp. *sylvestris* (L.) Hoffm. | #1100 | Russia, Karachay-Cherkessia Republic, valley of Marukha river, near a place called Morg-Syrt, 1790 m, 27 July 1929, *S.S.Nenjukov* *3020* (LE) | 43.52 41.45 0.963 km | ON180615 | ON584042, ON584043 | ON584113 | ON637456 | ON637354 | *Syl, Afroasiatic group, A* |
| *Anthriscus sylvestris* subsp. *sylvestris* (L.) Hoffm. | #1174 | Russia, Primorsky Krai, Nakhodka, the shore of Vostok Bay, Vostok Biological Station, 5 August 2006, *Chernyshev* (sample provided by A.A. Oskolski) | 42.88 132.73 0.400 km | ON180617 |  | ON584121 | ON637464 | ON637362 | *Syl, C* |
| *Anthriscus sylvestris* subsp. *sylvestris* (L.) Hoffm. | #1176 | Croatia, Oštarije above Karlobag, 26 July 1962, *Bedalov* (BM) | 44.53 15.17 0.725 km | ON180625 |  | ON584143 | ON637486 | ON637384 | *Syl, E* |
| *Anthriscus sylvestris* subsp. *sylvestris* (L.) Hoffm. | #1182 | Japan, Honshu, Shiga pref., Shimono, Yokaichi-shi, along the Echi River, 12 May 1990, *S.Tsugaru, G.Murata & T.Takahashi* *13158* (MO 4021782) | 35.12 136.21 1.054 km | ON180667 |  |  |  |  | *Syl* |
| *Anthriscus sylvestris* subsp. *sylvestris* (L.) Hoffm. | #1201 | Italy, Piedmont, Biella, 21 May 1926, *P.Fontana* *s.n.* (VAL) | 45.56 8.06 2.084 km | ON180622 |  |  |  |  | *Syl* |
| *Anthriscus sylvestris* subsp. *sylvestris* (L.) Hoffm. | #1202 | Japan, Honshu, Ishikawa pref., 1.2 km E from Ikenojomachi to Mitsutanimachi, Komatsu-shi, 220 m, 28 May 1986, *K.Deguchi, S.Tsugaru & M.Takeuchi* *7072* (MO 4011994) | 36.35 136.55 0.350 km | GQ379320* |  | ON584126 | ON637469 | ON637367 | *Syl, C* |
| *Anthriscus sylvestris* subsp. *sylvestris* (L.) Hoffm. | #1212 | Ethiopia, Oromia region, S face of Gara Mullata Mt., ca. 50 km due W of Harar, 9°12′N 41°46′E, c 2600 m, 24 May 1969, *J.J.F.E.De Wilde* *5067* (MO 2730033) | 9.24 41.73 0.652 km | ON180618 |  | ON584115 | ON637458 | ON637356 | *Syl, A* |
| *Anthriscus sylvestris* subsp. *sylvestris* (L.) Hoffm. | #1213 | Japan, Honshu, Miyagi Pref., Shiroishi-shi, Obara, 13 June 1970, *K.Sugawara et al.* (MO 3004767) | 37.96 140.56 0.617 km | ON180656 | ON584033 | ON584129 | ON637472 | ON637370 | *Syl, Afroasiatic group, C* |
| *Anthriscus sylvestris* subsp. *sylvestris* (L.) Hoffm. | #1215 | Tanzania, Arumeru distr., Mt. Meru, at end of vehicle track from Forestry Training Institute, 3°14′S 36°43′E, 2820 m, 11 January 1985, *R.E.Gereau* *1669* (MO 04639578) | -3.23 36.71 0.864 km | ON180537 |  |  |  |  | *Syl* |
| *Anthriscus sylvestris* subsp. *sylvestris* (L.) Hoffm. | #1216 | Japan, Honshu, Tōhoku region, Miyagi pref., Sendai, Aobayama, 50 m, 15 May 1968, *K.Sohma* *1008* (MO 1961769) | 38.25 140.84 1.003 km | ON180659 |  |  |  |  | *Syl* |
| *Anthriscus sylvestris* subsp. *sylvestris* (L.) Hoffm. | #1224 | China, Yunnan prov., eastern flank of the Lijang Range, 27°30′N, 11000 ft, Jun 1910, *G.Forrest* *5914* (E E00000103) | 27.20 100.15 14.000 km | ON180535 |  |  |  |  | *Syl* |
| *Anthriscus sylvestris* subsp. *sylvestris* (L.) Hoffm. | #1263 | France, Hérault dept., Minerve, place named Carrère, between Le Bouis and Boisset, 480 m, 3 June 1983, *J.-P.Reduron* *1983060301* (WA 0000071585) | 43.35 2.75 0.600 km | ON180660 |  |  |  |  | *Syl* |
| *Anthriscus sylvestris* subsp. *sylvestris* (L.) Hoffm. | #1285 | China, Yunnan prov., Yangbi Xian, W side of Cang mountains, vicinity of Shimenguang., 25°46′N 100°1′E, 3300-3600 m, 24 June 1984, *Sino-American Botanical Expedition* *413* (E E00183154) | 25.77 100.02 0.435 km | ON180654 |  | ON584127 | ON637470 | ON637368 | *Syl, C* |
| *Anthriscus sylvestris* subsp. *sylvestris* (L.) Hoffm. | #1305 | France, Pyrénées-Orientales Dept., Cerdagne, gorges of Llo, 1500 m, 15 July 1919, *F.Sennen* *3693* (E E00011020) | 42.45 2.07 0.410 km | ON180630 |  | ON584146 | ON637489 | ON637387 | *Syl, E* |
| *Anthriscus sylvestris* subsp. *sylvestris* (L.) Hoffm. | #1308 | Algeria, Blida prov., Chréa, 1500 m, 15 June 1975, *P.H.Davis* *59135* (E E00183169) | 36.43 2.88 0.939 km | ON180637 |  | ON584133 | ON637476 | ON637374 | *Syl, F* |
| *Anthriscus sylvestris* subsp. *sylvestris* (L.) Hoffm. | #1309 | Algeria, Tissemsilt prov., Théniet El Had National Park, Théniet El Had to Rond-Point des Cèdres, 1450-1500 m, 31 May 1975, *P.H.Davis* *58449* (E E00183168) | 35.87 1.94 0.350 km | ON180638 |  | ON584134 | ON637477 | ON637375 | *Syl, F* |
| *Anthriscus sylvestris* subsp. *sylvestris* (L.) Hoffm. | #1368 | China, Gansu prov., 25 km W from Pingliang town, Kongtong Mountains, 10 March 1957, *M.P.Petrov* (LE) | 35.55 106.48 1.503 km | ON180651 | ON584037 | ON584123 | ON637466 | ON637364 | *Syl, Afroasiatic group, C* |
| *Anthriscus sylvestris* subsp. *sylvestris* (L.) Hoffm. | #1369 | Russia, Kabardino-Balkaria, Chereksky distr., near Bezengi, left bank of Cherek-Bezengiyskiy river, 10 km down from Bezengi village, 1800-2000 m, 27 July 1988, *T.Popova* *23* (LE) | 43.14 43.17 2.337 km | ON180616 |  | ON584114 | ON637457 | ON637355 | *Syl, A* |
| *Anthriscus sylvestris* subsp. *sylvestris* (L.) Hoffm. | #1391 | Georgia, Racha-Lechkhumi and Kvemo Svaneti region, Oni distr., gorge of the Glola river below Mamisoni Pass, 22 July 1971, *E.E.Gogina* (LE) | 42.71 43.79 0.800 km | ON180648 |  | ON584119 | ON637462 | ON637360 | *Syl, A* |
| *Anthriscus sylvestris* subsp. *sylvestris* (L.) Hoffm. | #1428 | Algeria, Tell Atlas, Djurdjura range, near Tikjda, 1500 m, 3 June 1971, *P.H.Davis* *53083* (E E00183167) | 36.45 4.14 1.238 km | ON180636 | ON584027 | ON584132 | ON637475 | ON637373 | *Syl, North African group, F* |
| *Anthriscus sylvestris* subsp. *sylvestris* (L.) Hoffm. | #1429 | Yemen, Ta'izz gov., Sabir Al Mawadim distr., Maḩzaf, 13 December 1983, *Gordon* *504* (E E00183166) | 13.53 44.04 0.770 km | ON180649 |  | ON584120 | ON637463 | ON637361 | *Syl, A* |
| *Anthriscus sylvestris* subsp. *sylvestris* (L.) Hoffm. | #1430 | South Africa, Eastern Cape, Barkly East, valley of Bell river near Rhodes, 6200 ft, 28 November 1971, *O.M.Hilliard* *5220* (E E00183158) | -30.80 27.96 0.842 km | ON180657 |  |  |  |  | *Syl* |
| *Anthriscus sylvestris* subsp. *sylvestris* (L.) Hoffm. | #1432 | Kenya, Mt. Kenya, Kathita River (NE sector), 9800 ft, 4 August 1949, *E.A.C.L.E.Schelpe* *2677* (E E00183156) | -0.03 37.47 0.877 km | ON180647 | ON584029 | ON584118 | ON637461 | ON637359 | *Syl, Afroasiatic group, A* |
| *Anthriscus sylvestris* subsp. *sylvestris* (L.) Hoffm. | #1435 | China, Quinghai prov., Yushu Zang Aut. Pref., Jiangxi Gou, E of Jiangxi Forest Station on E side of the Zi Qu, SE of Mozhong, 32°4′N 97°2′E, 28 August 1996, *T.N.Ho, B.Bartholomew, M.F.Watson & M.Gilbert* *2595* (E E00183153) | 32.07 97.03 0.851 km | ON180655 |  | ON584128 | ON637471 | ON637369 | *Syl, C* |
| *Anthriscus sylvestris* subsp. *sylvestris* (L.) Hoffm. | #1437 | China, Sichuan prov., Zheduo Pass, above Kangding, 3500 m, 26 September 1991, *D.Chamberlain, D.Knott, Pu Fating & Wang Pingli* *CEE 386* (E E00000773) | 29.97 101.85 1.562 km | ON180665 |  |  |  |  | *Syl* |
| *Anthriscus sylvestris* subsp. *sylvestris* (L.) Hoffm. | #1441 | Norway, Finnmark county, Berlevåg municipality, Kongsfjord, 18 July 1975, *R.F.Thorne & R.Alava* *45926* (E E00183139) | 70.72 29.32 0.428 km | ON180642 | ON584023 | ON584151 | ON637494 | ON637392 | *Syl, European lowland group, E* |
| *Anthriscus sylvestris* subsp. *sylvestris* (L.) Hoffm. | #1443 | Finland, Regio aboensis, inner archipelago of Turku, Nauvo, Nauvo mainland, north-facing slope of Klockarberget hill, 21 June 1971, *S.Hinneri & U.Laine* (E E00183137) | 0.00 0.00 0.000 km | ON180620 | ON584024, ON584025 | ON584141 | ON637484 | ON637382 | *Syl, European lowland group, E* |
| *Anthriscus sylvestris* subsp. *sylvestris* (L.) Hoffm. | #1445 | China, Yunnan prov., Diqing pref., Xia Geza, 39 km N from Zhongdian, near Liutung He river, 28°4′20″N 99°46′15″E, 3150 m, 26 May 1993, *B.Aldén, J.C.M.Alexander, D.G.Long, R.J.D.McBeath, H.J.Noltie & M.F.Watson* *KEG 281* (E E00000816) | 28.21 99.74 1.320 km | ON180652 |  | ON584124 | ON637467 | ON637365 | *Syl, C* |
| *Anthriscus sylvestris* subsp. *sylvestris* (L.) Hoffm. | #1448 | Spain, Madrid, El Paular, 1170 m, 29 July 1980, *F.Fernandez Gonzalez* (SANT) | 40.89 -3.89 0.410 km | ON180631 |  | ON584147 | ON637490 | ON637388 | *Syl, E* |
| *Anthriscus sylvestris* subsp. *sylvestris* (L.) Hoffm. | #1449 | Spain, Galicia, Ourense prov., Celanova, río Orille, 360 m, 19 May 2000, *I.Pulgar* (SANT) | 42.16 -7.94 1.015 km | ON180633 |  | ON584149 | ON637492 | ON637390 | *Syl, E* |
| *Anthriscus sylvestris* subsp. *sylvestris* (L.) Hoffm. | #1452 | Spain, Aragon, Teruel prov., Peracense, Monte de San Gines, 1600 m, 21 July 1995, *C.Fabregat & S.López Udías* (VAL) | 40.62 -1.47 0.299 km | ON180619 | ON584013, ON584014 | ON584139 | ON637482 | ON637380 | *Syl, European lowland group, E* |
| *Anthriscus sylvestris* subsp. *sylvestris* (L.) Hoffm. | #1455 | Spain, Catalonia, Tarragona prov., Ports de Tortosa-Beseit, 1200 m, Jun 1979, *J.Mansanet & G.Mateo* *79/404* (VAL) | 0.00 0.00 0.000 km | ON180632 |  | ON584148 | ON637491 | ON637389 | *Syl, E* |
| *Anthriscus sylvestris* subsp. *sylvestris* (L.) Hoffm. | #1457 | Spain, Comunidad Valenciana, Castellón prov., Villafranca del Cid, near Puebla de Ballestar, 1000 m, 19 June 1993, *C.Fabregat & S.López Udías* (VAL) | 40.43 -0.26 0.892 km | ON180666 |  |  |  |  | *Syl* |
| *Anthriscus sylvestris* subsp. *sylvestris* (L.) Hoffm. | #1462 | Spain, Castilla y León, Ávila prov., Villafranca de la Sierra, 23 June 1974, *G.A.López González & E.Valdes-Bermejo* *1652* (VAL) | 40.50 -5.23 0.404 km | ON180621 |  | ON584140 | ON637483 | ON637381 | *Syl, E* |
| *Anthriscus sylvestris* subsp. *sylvestris* (L.) Hoffm. | #1470 | Slovenia, near Ljubljana, 300 m, 11 March 1913, *A.Paulin* *949* (BM) | 46.06 14.51 5.995 km | ON180658 |  |  |  |  | *Syl* |
| *Anthriscus sylvestris* subsp. *sylvestris* (L.) Hoffm. | #1485 | Romania, Transylvania, Covasna county, near Sfântu Gheorghe, 13 June 1973, *M.Danciu* *3466* (BM) | 45.87 25.79 3.086 km | ON180624 |  | ON584142 | ON637485 | ON637383 | *Syl, E* |
| *Anthriscus sylvestris* subsp. *sylvestris* (L.) Hoffm. | #1488 | Uganda, Rwenzori Mountains, Nyamugasani valley, Jan 1935, *P.M.Synge* *1488* (BM BM000998971) | 0.14 29.93 3.250 km | ON180627 |  |  |  |  | *Syl* |
| *Anthriscus sylvestris* subsp. *sylvestris* (L.) Hoffm. | #1492 | Morocco, Ifrane prov., Middle Atlas, 5 km from Ifrane, Azrou, 1600 m, 6 June 1971, *G.Bocquet* *10360* (BM BM000998967) | 33.50 -5.15 2.750 km | ON180639 |  | ON584135 | ON637478 | ON637376 | *Syl, F* |
| *Anthriscus sylvestris* subsp. *sylvestris* (L.) Hoffm. | #1494 | Kazakhstan, Almaty, 2 June 1907, *N.D.Sokalsky* (BM) | 43.25 76.89 6.460 km | ON180628 |  | ON584130 | ON637473 | ON637371 | *Syl, C* |
| *Anthriscus sylvestris* subsp. *sylvestris* (L.) Hoffm. | #1501 | China, Hubei prov., 1885 1888, *A.Henry* *6992* (BM BM000537151) | 31.20 112.30 450.000 km | ON180653 |  | ON584125 | ON637468 | ON637366 | *Syl, C* |
| *Anthriscus sylvestris* subsp. *sylvestris* (L.) Hoffm. | #1505 | Algeria, Tlemcen prov., El-Ourit Waterfalls E of Tlemcen, 700-800 m, 23 April 1971, *P.H.Davis* *51480* (BM BM000537140) | 34.86 -1.27 0.401 km | ON180664 |  |  |  |  | *Syl* |
| *Anthriscus sylvestris* subsp. *sylvestris* (L.) Hoffm. | #1512 | Italy, Veneto, Verona prov., Monte Baldo, Val Trovai, 1000-1500 m, 12 July 1904, *G.Rigo* *594* (BM BM000537137) | 45.70 10.80 0.950 km | ON180645 |  | ON584155 | ON637499 | ON637397 | *Syl, E* |
| *Anthriscus sylvestris* subsp. *sylvestris* (L.) Hoffm. | #1748 | Poland, Szlachtowa village, "Las Na Brzegu" forest, Nov 2007, *Ł.Banasiak* *s.n.* | 49.41 20.52 2.413 km | ON064025 | ON584017, ON584018 |  |  |  | *Syl, European lowland group* |
| *Anthriscus sylvestris* subsp. *sylvestris* (L.) Hoffm. | #1750 | Poland, Szlachtowa village, "Las Na Brzegu" forest, 18 September 2007, *Ł.Banasiak* *s.n.* (WA 0000071569) | 49.41 20.52 2.413 km | ON064026 | ON584015, ON584016 |  |  |  | *Syl, Intermediate* |
| *Anthriscus sylvestris* subsp. *sylvestris* (L.) Hoffm. | #2301 | Ethiopia, Oromia region, W slopes of Boruluccu, along road to Gobesa, 25 km SE from Assela, 6 December 1965, *J.J.F.E.De Wilde* *9189* (MO 2265792) | 7.80 39.29 11.512 km | ON180536 |  | ON584117 | ON637460 | ON637358 | *Syl, A* |
| *Anthriscus sylvestris* subsp. *sylvestris* (L.) Hoffm. | #2314 | Russia, Stavropol Krai, Mt. Mashuk near Pyatigorsk, 25 June 1896, *D.I.Litwinow* (LE) | 44.05 43.09 1.016 km | ON180623 |  |  |  |  | *Syl* |
| *Anthriscus sylvestris* subsp. *sylvestris* (L.) Hoffm. | #2315 | Georgia, South Ossetia, Chaparukhi gorge, Zandzhiket near Lbir, 1900 m, 10 August 1930, *E.Busch & N.Busch* (LE) | 42.40 44.23 3.488 km | ON180663 |  |  |  |  | *Syl* |
| *Anthriscus sylvestris* subsp. *sylvestris* (L.) Hoffm. | #2547 | Poland, 53.0287°N 18.6934°E, 27 August 2018, *M.Piwczyński* (WA 0000165370) |  | PP110846 |  |  |  |  | *Syl* |
| *Anthriscus sylvestris* subsp. *sylvestris* (L.) Hoffm. | #2548 | Poland, 52.1052°N 19.4832°E, 99 m, 20 May 2020, *P.Trzeciak & M.Piwczyński* (WA 0000165371) |  | PP110847 |  |  |  |  | *Syl* |
| *Anthriscus sylvestris* subsp. *sylvestris* (L.) Hoffm. | #2549 | Poland, 51.9328°N 19.4335°E, 134 m, 20 May 2020, *P.Trzeciak & M.Piwczyński* (WA 0000165372) |  | PP110848 |  |  |  |  | *Syl* |
| *Anthriscus sylvestris* subsp. *sylvestris* (L.) Hoffm. | #2550 | Poland, 51.8982°N 19.3663°E, 162 m, 20 May 2020, *P.Trzeciak & M.Piwczyński* (WA 0000165373) |  | PP110849 |  |  |  |  | *Syl* |
| *Anthriscus sylvestris* subsp. *sylvestris* (L.) Hoffm. | #2551 | Poland, 51.8974°N 19.3645°E, 164 m, 20 May 2020, *P.Trzeciak & M.Piwczyński* (WA 0000165374) |  | PP110850 |  |  |  |  | *Syl* |
| *Anthriscus sylvestris* subsp. *sylvestris* (L.) Hoffm. | #2552 | Poland, 51.8937°N 19.3647°E, 167 m, 20 May 2020, *P.Trzeciak & M.Piwczyński* (WA 0000165375) |  | PP110851 |  |  |  |  | *Syl* |
| *Anthriscus sylvestris* subsp. *sylvestris* (L.) Hoffm. | #2553 | Poland, 51.9261°N 20.3682°E, 165 m, 20 May 2020, *P.Trzeciak & M.Piwczyński* (WA 0000165376) |  | PP110852 |  |  |  |  | *Syl* |
| *Anthriscus sylvestris* subsp. *sylvestris* (L.) Hoffm. | #2554 | Poland, 51.9264°N 20.3676°E, 164 m, 20 May 2020, *P.Trzeciak & M.Piwczyński* (WA 0000165377) |  | PP110853 |  |  |  |  | *Syl* |
| *Anthriscus sylvestris* subsp. *sylvestris* (L.) Hoffm. | #2555 | Poland, 51.8755°N 20.832°E, 147 m, 20 May 2020, *P.Trzeciak & M.Piwczyński* (WA 0000165378) |  | PP110854 |  |  |  |  | *Syl* |
| *Anthriscus sylvestris* subsp. *sylvestris* (L.) Hoffm. | #2556 | Poland, 51.5814°N 20.9986°E, 151 m, 20 May 2020, *P.Trzeciak & M.Piwczyński* (WA 0000165379) |  | PP110855 |  |  |  |  | *Syl* |
| *Anthriscus sylvestris* subsp. *sylvestris* (L.) Hoffm. | #2557 | Poland, 51.5083°N 21.1002°E, 137 m, 20 May 2020, *P.Trzeciak & M.Piwczyński* (WA 0000165380) |  | PP110856 |  |  |  |  | *Syl* |
| *Anthriscus sylvestris* subsp. *sylvestris* (L.) Hoffm. | #2558 | Poland, 50.7533°N 20.6144°E, 235 m, 20 May 2020, *P.Trzeciak & M.Piwczyński* (WA 0000165381) |  | PP110857 |  |  |  |  | *Syl* |
| *Anthriscus sylvestris* subsp. *sylvestris* (L.) Hoffm. | #2559 | Poland, 50.3665°N 21.0529°E, 171 m, 20 May 2020, *P.Trzeciak & M.Piwczyński* (WA 0000165382) |  | PP110858 |  |  |  |  | *Syl* |
| *Anthriscus sylvestris* subsp. *sylvestris* (L.) Hoffm. | #2560 | Poland, 49.3386°N 20.8241°E, 441 m, 21 May 2020, *P.Trzeciak & M.Piwczyński* (WA 0000165383) |  | PP110859 |  |  |  |  | *Syl* |
| *Anthriscus sylvestris* subsp. *sylvestris* (L.) Hoffm. | #2561 | Poland, 49.3366°N 20.83°E, 451 m, 21 May 2020, *P.Trzeciak & M.Piwczyński* (WA 0000165384) |  | PP110860 |  |  |  |  | *Syl* |
| *Anthriscus sylvestris* subsp. *sylvestris* (L.) Hoffm. | #2562 | Poland, 49.4124°N 20.7673°E, 435 m, 21 May 2020, *P.Trzeciak & M.Piwczyński* (WA 0000165385) |  | PP110861 |  |  |  |  | *Syl* |
| *Anthriscus sylvestris* subsp. *sylvestris* (L.) Hoffm. | #2563 | Poland, 49.425°N 20.7881°E, 487 m, 21 May 2020, *P.Trzeciak & M.Piwczyński* (WA 0000165386) |  | PP110862 |  |  |  |  | *Syl* |
| *Anthriscus sylvestris* subsp. *sylvestris* (L.) Hoffm. | #2564 | Poland, 49.4252°N 20.7888°E, 483 m, 21 May 2020, *P.Trzeciak & M.Piwczyński* (WA 0000165387) |  | PP110863 |  |  |  |  | *Syl* |
| *Anthriscus sylvestris* subsp. *sylvestris* (L.) Hoffm. | #2565 | Poland, 49.4331°N 20.7176°E, 390 m, 21 May 2020, *P.Trzeciak & M.Piwczyński* (WA 0000165388) |  | PP110864 |  |  |  |  | *Syl* |
| *Anthriscus sylvestris* subsp. *sylvestris* (L.) Hoffm. | #2566 | Poland, 49.4458°N 20.7097°E, 373 m, 21 May 2020, *P.Trzeciak & M.Piwczyński* (WA 0000165389) |  | PP110865 |  |  |  |  | *Syl* |
| *Anthriscus sylvestris* subsp. *sylvestris* (L.) Hoffm. | #2567 | Poland, 49.4576°N 20.6939°E, 373 m, 21 May 2020, *P.Trzeciak & M.Piwczyński* (WA 0000165390) |  | PP110866 |  |  |  |  | *Syl* |
| *Anthriscus sylvestris* subsp. *sylvestris* (L.) Hoffm. | #2568 | Poland, 49.4583°N 20.6939°E, 375 m, 21 May 2020, *P.Trzeciak & M.Piwczyński* (WA 0000165391) |  | PP110867 |  |  |  |  | *Syl* |
| *Anthriscus sylvestris* subsp. *sylvestris* (L.) Hoffm. | #2569 | Poland, 49.4682°N 20.6964°E, 359 m, 21 May 2020, *P.Trzeciak & M.Piwczyński* (WA 0000165392) |  | PP110868 |  |  |  |  | *Syl* |
| *Anthriscus sylvestris* subsp. *sylvestris* (L.) Hoffm. | #2570 | Poland, 49.5774°N 20.675°E, 323 m, 21 May 2020, *P.Trzeciak & M.Piwczyński* (WA 0000165393) |  | PP110869 |  |  |  |  | *Syl* |
| *Anthriscus sylvestris* subsp. *sylvestris* (L.) Hoffm. | #2571 | Poland, 49.5773°N 20.6755°E, 326 m, 21 May 2020, *P.Trzeciak & M.Piwczyński* (WA 0000165394) |  | PP110870 |  |  |  |  | *Syl* |
| *Anthriscus sylvestris* subsp. *sylvestris* (L.) Hoffm. | #2572 | Poland, 49.5497°N 20.7753°E, 382 m, 21 May 2020, *P.Trzeciak & M.Piwczyński* (WA 0000165395) |  | PP110871 |  |  |  |  | *Syl* |
| *Anthriscus sylvestris* subsp. *sylvestris* (L.) Hoffm. | #2573 | Poland, 49.5317°N 20.8058°E, 436 m, 21 May 2020, *P.Trzeciak & M.Piwczyński* (WA 0000165396) |  | PP110872 |  |  |  |  | *Syl* |
| *Anthriscus sylvestris* subsp. *sylvestris* (L.) Hoffm. | #2574 | Poland, 49.4486°N 20.9525°E, 743 m, 22 May 2020, *P.Trzeciak & M.Piwczyński* (WA 0000165397) |  | PP110873 |  |  |  |  | *Syl* |
| *Anthriscus sylvestris* subsp. *sylvestris* (L.) Hoffm. | #2575 | Poland, 49.4273°N 21.0226°E, 620 m, 22 May 2020, *P.Trzeciak & M.Piwczyński* (WA 0000165398) |  | PP110874 |  |  |  |  | *Syl* |
| *Anthriscus sylvestris* subsp. *sylvestris* (L.) Hoffm. | #2576 | Poland, 49.3836°N 21.0616°E, 621 m, 22 May 2020, *P.Trzeciak & M.Piwczyński* (WA 0000165399) |  | PP110875 |  |  |  |  | *Syl* |
| *Anthriscus sylvestris* subsp. *sylvestris* (L.) Hoffm. | #2577 | Poland, 49.3747°N 21.1018°E, 687 m, 22 May 2020, *P.Trzeciak & M.Piwczyński* (WA 0000165400) |  | PP110835 |  |  |  |  | *Nit* |
| *Anthriscus sylvestris* subsp. *sylvestris* (L.) Hoffm. | #2578 | Poland, 49.3759°N 21.0913°E, 665 m, 22 May 2020, *P.Trzeciak & M.Piwczyński* (WA 0000165401) |  | PP110876 |  |  |  |  | *Syl* |
| *Anthriscus sylvestris* subsp. *sylvestris* (L.) Hoffm. | #2579 | Poland, 49.479°N 20.9238°E, 589 m, 22 May 2020, *P.Trzeciak & M.Piwczyński* (WA 0000165402) |  | PP110877 |  |  |  |  | *Syl* |
| *Anthriscus sylvestris* subsp. *sylvestris* (L.) Hoffm. | #2580 | Poland, 49.6495°N 20.5932°E, 514 m, 22 May 2020, *P.Trzeciak & M.Piwczyński* (WA 0000165403) |  | PP110878 |  |  |  |  | *Syl* |
| *Anthriscus sylvestris* subsp. *sylvestris* (L.) Hoffm. | #2581 | Poland, 49.6812°N 20.4721°E, 537 m, 22 May 2020, *P.Trzeciak & M.Piwczyński* (WA 0000165404) |  | PP110879 |  |  |  |  | *Syl* |
| *Anthriscus sylvestris* subsp. *sylvestris* (L.) Hoffm. | #2582 | Poland, 49.7214°N 20.3022°E, 500 m, 22 May 2020, *P.Trzeciak & M.Piwczyński* (WA 0000165405) |  | PP110880 |  |  |  |  | *Syl* |
| *Anthriscus sylvestris* subsp. *sylvestris* (L.) Hoffm. | #2583 | Poland, 49.9°N 19.6869°E, 268 m, 22 May 2020, *P.Trzeciak & M.Piwczyński* (WA 0000165406) |  | PP110881 |  |  |  |  | *Syl* |
| *Anthriscus sylvestris* subsp. *sylvestris* (L.) Hoffm. | #2584 | Poland, 50.5753°N 17.8904°E, 203 m, 23 May 2020, *P.Trzeciak & M.Piwczyński* (WA 0000165407) |  | PP110882 |  |  |  |  | *Syl* |
| *Anthriscus sylvestris* subsp. *sylvestris* (L.) Hoffm. | #2585 | Poland, 50.5775°N 17.9033°E, 192 m, 23 May 2020, *P.Trzeciak & M.Piwczyński* (WA 0000165408) |  | PP110883 |  |  |  |  | *Syl* |
| *Anthriscus sylvestris* subsp. *sylvestris* (L.) Hoffm. | #2586 | Poland, 50.6297°N 17.9174°E, 167 m, 23 May 2020, *P.Trzeciak & M.Piwczyński* (WA 0000165409) |  | PP110884 |  |  |  |  | *Syl* |
| *Anthriscus sylvestris* subsp. *sylvestris* (L.) Hoffm. | #2587 | Poland, 50.8598°N 18.1945°E, 206 m, 23 May 2020, *P.Trzeciak & M.Piwczyński* (WA 0000165410) |  | PP110885 |  |  |  |  | *Syl* |
| *Anthriscus sylvestris* subsp. *sylvestris* (L.) Hoffm. | #2588 | Poland, 51.021°N 18.3984°E, 210 m, 23 May 2020, *P.Trzeciak & M.Piwczyński* (WA 0000165411) |  | PP110886 |  |  |  |  | *Syl* |
| *Anthriscus sylvestris* subsp. *sylvestris* (L.) Hoffm. | #2589 | Poland, 51.2609°N 18.5592°E, 184 m, 23 May 2020, *P.Trzeciak & M.Piwczyński* (WA 0000165412) |  | PP110887 |  |  |  |  | *Syl* |
| *Anthriscus sylvestris* subsp. *sylvestris* (L.) Hoffm. | #2590 | Poland, 50.1471°N 19.2996°E, 270 m, 7 June 2020, *P.Trzeciak & M.Piwczyński* (WA 0000165413) |  | PP110888 |  |  |  |  | *Syl* |
| *Anthriscus sylvestris* subsp. *sylvestris* (L.) Hoffm. | #2591 | Poland, 49.4186°N 20.5063°E, 506 m, 8 June 2020, *P.Trzeciak & M.Piwczyński* (WA 0000165414) |  | PP110889 |  |  |  |  | *Syl* |
| *Anthriscus sylvestris* subsp. *sylvestris* (L.) Hoffm. | #2592 | Poland, 49.4057°N 20.5409°E, 576 m, 8 June 2020, *P.Trzeciak & M.Piwczyński* (WA 0000165415) |  | PP110890 |  |  |  |  | *Syl* |
| *Anthriscus sylvestris* subsp. *sylvestris* (L.) Hoffm. | #2593 | Poland, 49.4032°N 20.5442°E, 659 m, 8 June 2020, *P.Trzeciak & M.Piwczyński* (WA 0000165416) |  | PP110891 |  |  |  |  | *Syl* |
| *Anthriscus sylvestris* subsp. *sylvestris* (L.) Hoffm. | #2594 | Poland, 49.4027°N 20.5439°E, 672 m, 8 June 2020, *P.Trzeciak & M.Piwczyński* (WA 0000165417) |  | PP110892 |  |  |  |  | *Syl* |
| *Anthriscus sylvestris* subsp. *sylvestris* (L.) Hoffm. | #2595 | Poland, 49.3978°N 20.5845°E, 667 m, 8 June 2020, *P.Trzeciak & M.Piwczyński* (WA 0000165418) |  | PP110893 |  |  |  |  | *Syl* |
| *Anthriscus sylvestris* subsp. *sylvestris* (L.) Hoffm. | #2596 | Poland, 49.3998°N 20.582°E, 649 m, 8 June 2020, *P.Trzeciak & M.Piwczyński* (WA 0000165419) |  | PP110894 |  |  |  |  | *Syl* |
| *Anthriscus sylvestris* subsp. *sylvestris* (L.) Hoffm. | #2597 | Poland, 49.4746°N 20.4201°E, 463 m, 8 June 2020, *P.Trzeciak & M.Piwczyński* (WA 0000165420) |  | PP110895 |  |  |  |  | *Syl* |
| *Anthriscus sylvestris* subsp. *sylvestris* (L.) Hoffm. | #2598 | Poland, 49.475°N 20.4205°E, 438 m, 8 June 2020, *P.Trzeciak & M.Piwczyński* (WA 0000165421) |  | PP110896 |  |  |  |  | *Syl* |
| *Anthriscus sylvestris* subsp. *sylvestris* (L.) Hoffm. | #2599 | Poland, 49.5158°N 20.5372°E, 417 m, 8 June 2020, *P.Trzeciak & M.Piwczyński* (WA 0000165422) |  | PP110897 |  |  |  |  | *Syl* |
| *Anthriscus sylvestris* subsp. *sylvestris* (L.) Hoffm. | #2600 | Poland, 49.5162°N 20.5369°E, 426 m, 8 June 2020, *P.Trzeciak & M.Piwczyński* (WA 0000165423) |  | PP110898 |  |  |  |  | *Syl* |
| *Anthriscus sylvestris* subsp. *sylvestris* (L.) Hoffm. | #2601 | Poland, 49.4547°N 20.4226°E, 490 m, 9 June 2020, *P.Trzeciak & M.Piwczyński* (WA 0000165424) |  | PP110899 |  |  |  |  | *Syl* |
| *Anthriscus sylvestris* subsp. *sylvestris* (L.) Hoffm. | #2602 | Poland, 49.4524°N 20.4212°E, 458 m, 9 June 2020, *P.Trzeciak & M.Piwczyński* (WA 0000165425) |  | PP110900 |  |  |  |  | *Syl* |
| *Anthriscus sylvestris* subsp. *sylvestris* (L.) Hoffm. | #2603 | Poland, 49.4621°N 20.4277°E, 424 m, 9 June 2020, *P.Trzeciak & M.Piwczyński* (WA 0000165426) |  | PP110901 |  |  |  |  | *Syl* |
| *Anthriscus sylvestris* subsp. *sylvestris* (L.) Hoffm. | #2604 | Poland, 49.6009°N 20.3519°E, 541 m, 9 June 2020, *P.Trzeciak & M.Piwczyński* (WA 0000165427) |  | PP110902 |  |  |  |  | *Syl* |
| *Anthriscus sylvestris* subsp. *sylvestris* (L.) Hoffm. | #2605 | Poland, 49.6288°N 20.3361°E, 766 m, 9 June 2020, *P.Trzeciak & M.Piwczyński* (WA 0000165428) |  | PP110903 |  |  |  |  | *Syl* |
| *Anthriscus sylvestris* subsp. *sylvestris* (L.) Hoffm. | #2606 | Poland, 49.6318°N 20.3377°E, 771 m, 9 June 2020, *P.Trzeciak & M.Piwczyński* (WA 0000165429) |  | PP110904 |  |  |  |  | *Syl* |
| *Anthriscus sylvestris* subsp. *sylvestris* (L.) Hoffm. | #2607 | Poland, 49.6349°N 20.3768°E, 763 m, 9 June 2020, *P.Trzeciak & M.Piwczyński* (WA 0000165430) |  | PP110905 |  |  |  |  | *Syl* |
| *Anthriscus sylvestris* subsp. *sylvestris* (L.) Hoffm. | #2608 | Poland, 49.6022°N 20.4633°E, 511 m, 9 June 2020, *P.Trzeciak & M.Piwczyński* (WA 0000165431) |  | PP110906 |  |  |  |  | *Syl* |
| *Anthriscus sylvestris* subsp. *sylvestris* (L.) Hoffm. | #2609 | Poland, 49.622°N 20.4523°E, 575 m, 9 June 2020, *P.Trzeciak & M.Piwczyński* (WA 0000165432) |  | PP110907 |  |  |  |  | *Syl* |
| *Anthriscus sylvestris* subsp. *sylvestris* (L.) Hoffm. | #2610 | Poland, 49.6117°N 20.3466°E, 606 m, 9 June 2020, *P.Trzeciak & M.Piwczyński* (WA 0000165433) |  | PP110908 |  |  |  |  | *Syl* |
| *Anthriscus sylvestris* subsp. *sylvestris* (L.) Hoffm. | #2611 | Poland, 49.6117°N 20.3472°E, 597 m, 9 June 2020, *P.Trzeciak & M.Piwczyński* (WA 0000165434) |  | PP110909 |  |  |  |  | *Syl* |
| *Anthriscus sylvestris* subsp. *sylvestris* (L.) Hoffm. | #2612 | Poland, 49.419°N 20.4565°E, 444 m, 9 June 2020, *P.Trzeciak & M.Piwczyński* (WA 0000165435) |  | PP110910 |  |  |  |  | *Syl* |
| *Anthriscus sylvestris* subsp. *sylvestris* (L.) Hoffm. | #2613 | Poland, 50.9767°N 19.1669°E, 220 m, 10 June 2020, *P.Trzeciak & M.Piwczyński* (WA 0000165436) |  | PP110911 |  |  |  |  | *Syl* |
| *Anthriscus sylvestris* subsp. *sylvestris* (L.) Hoffm. | #2614 | Poland, 51.0178°N 19.1623°E, 207 m, 10 June 2020, *P.Trzeciak & M.Piwczyński* (WA 0000165437) |  | PP110912 |  |  |  |  | *Syl* |
| *Anthriscus sylvestris* subsp. *sylvestris* (L.) Hoffm. | #2615 | Poland, 51.3745°N 19.1528°E, 169 m, 10 June 2020, *P.Trzeciak & M.Piwczyński* (WA 0000165438) |  | PP110913 |  |  |  |  | *Syl* |
| *Anthriscus sylvestris* subsp. *sylvestris* (L.) Hoffm. | #2616 | Poland, 50.8298°N 15.5437°E, 561 m, 20 June 2020, *P.Trzeciak & M.Piwczyński* (WA 0000165439) |  | PP110914 |  |  |  |  | *Syl* |
| *Anthriscus sylvestris* subsp. *sylvestris* (L.) Hoffm. | #2617 | Poland, 50.8433°N 15.5454°E, 600 m, 20 June 2020, *P.Trzeciak & M.Piwczyński* (WA 0000165440) |  | PP110915 |  |  |  |  | *Syl* |
| *Anthriscus sylvestris* subsp. *sylvestris* (L.) Hoffm. | #2618 | Poland, 50.893°N 15.3723°E, 533 m, 20 June 2020, *P.Trzeciak & M.Piwczyński* (WA 0000165441) |  | PP110916 |  |  |  |  | *Syl* |
| *Anthriscus sylvestris* subsp. *sylvestris* (L.) Hoffm. | #2619 | Poland, 50.9195°N 15.3442°E, 447 m, 20 June 2020, *P.Trzeciak & M.Piwczyński* (WA 0000165442) |  | PP110917 |  |  |  |  | *Syl* |
| *Anthriscus sylvestris* subsp. *sylvestris* (L.) Hoffm. | #2620 | Poland, 50.9314°N 15.3833°E, 456 m, 20 June 2020, *P.Trzeciak & M.Piwczyński* (WA 0000165443) |  | PP110918 |  |  |  |  | *Syl* |
| *Anthriscus sylvestris* subsp. *sylvestris* (L.) Hoffm. | #2621 | Poland, 50.9288°N 15.4457°E, 458 m, 20 June 2020, *P.Trzeciak & M.Piwczyński* (WA 0000165444) |  | PP110919 |  |  |  |  | *Syl* |
| *Anthriscus sylvestris* subsp. *sylvestris* (L.) Hoffm. | #2622 | Poland, 50.9139°N 15.4946°E, 510 m, 20 June 2020, *P.Trzeciak & M.Piwczyński* (WA 0000165445) |  | PP110920 |  |  |  |  | *Syl* |
| *Anthriscus sylvestris* subsp. *sylvestris* (L.) Hoffm. | #2623 | Poland, 50.895°N 15.5063°E, 513 m, 20 June 2020, *P.Trzeciak & M.Piwczyński* (WA 0000165446) |  | PP110921 |  |  |  |  | *Syl* |
| *Anthriscus sylvestris* subsp. *sylvestris* (L.) Hoffm. | #2624 | Poland, 50.8819°N 15.5528°E, 545 m, 20 June 2020, *P.Trzeciak & M.Piwczyński* (WA 0000165447) |  | PP110922 |  |  |  |  | *Syl* |
| *Anthriscus sylvestris* subsp. *sylvestris* (L.) Hoffm. | #2625 | Poland, 50.8465°N 15.6114°E, 380 m, 21 June 2020, *P.Trzeciak & M.Piwczyński* (WA 0000165448) |  | PP110923 |  |  |  |  | *Syl* |
| *Anthriscus sylvestris* subsp. *sylvestris* (L.) Hoffm. | #2626 | Poland, 50.8355°N 15.6704°E, 356 m, 21 June 2020, *P.Trzeciak & M.Piwczyński* (WA 0000165449) |  | PP110924 |  |  |  |  | *Syl* |
| *Anthriscus sylvestris* subsp. *sylvestris* (L.) Hoffm. | #2627 | Poland, 50.8143°N 15.7553°E, 428 m, 21 June 2020, *P.Trzeciak & M.Piwczyński* (WA 0000165450) |  | PP110925 |  |  |  |  | *Syl* |
| *Anthriscus sylvestris* subsp. *sylvestris* (L.) Hoffm. | #2628 | Poland, 50.7757°N 15.7804°E, 597 m, 21 June 2020, *P.Trzeciak & M.Piwczyński* (WA 0000165451) |  | PP110926 |  |  |  |  | *Syl* |
| *Anthriscus sylvestris* subsp. *sylvestris* (L.) Hoffm. | #2629 | Poland, 50.7905°N 15.8074°E, 474 m, 21 June 2020, *P.Trzeciak & M.Piwczyński* (WA 0000165452) |  | PP110927 |  |  |  |  | *Syl* |
| *Anthriscus sylvestris* subsp. *sylvestris* (L.) Hoffm. | #2630 | Poland, 50.7893°N 15.8645°E, 580 m, 21 June 2020, *P.Trzeciak & M.Piwczyński* (WA 0000165453) |  | PP110928 |  |  |  |  | *Syl* |
| *Anthriscus sylvestris* subsp. *sylvestris* (L.) Hoffm. | #2631 | Poland, 50.7611°N 15.8686°E, 730 m, 21 June 2020, *P.Trzeciak & M.Piwczyński* (WA 0000165454) |  | PP110929 |  |  |  |  | *Syl* |
| *Anthriscus sylvestris* subsp. *sylvestris* (L.) Hoffm. | #2632 | Poland, 50.7378°N 15.8703°E, 675 m, 21 June 2020, *P.Trzeciak & M.Piwczyński* (WA 0000165455) |  | PP110930 |  |  |  |  | *Syl* |
| *Anthriscus sylvestris* subsp. *sylvestris* (L.) Hoffm. | #2633 | Poland, 50.7627°N 15.8677°E, 735 m, 21 June 2020, *P.Trzeciak & M.Piwczyński* (WA 0000165456) |  | PP110931 |  |  |  |  | *Syl* |
| *Anthriscus sylvestris* subsp. *sylvestris* (L.) Hoffm. | #2634 | Poland, 50.8461°N 15.786°E, 384 m, 21 June 2020, *P.Trzeciak & M.Piwczyński* (WA 0000165457) |  | PP110932 |  |  |  |  | *Syl* |
| *Anthriscus sylvestris* subsp. *sylvestris* (L.) Hoffm. | #2635 | Poland, 50.9256°N 15.7697°E, 364 m, 22 June 2020, *P.Trzeciak & M.Piwczyński* (WA 0000165458) |  | PP110933 |  |  |  |  | *Syl* |
| *Anthriscus sylvestris* subsp. *sylvestris* (L.) Hoffm. | #2636 | Poland, 50.9525°N 15.8099°E, 532 m, 22 June 2020, *P.Trzeciak & M.Piwczyński* (WA 0000165459) |  | PP110934 |  |  |  |  | *Syl* |
| *Anthriscus sylvestris* subsp. *sylvestris* (L.) Hoffm. | #2637 | Poland, 50.9698°N 15.8198°E, 532 m, 22 June 2020, *P.Trzeciak & M.Piwczyński* (WA 0000165460) |  | PP110935 |  |  |  |  | *Syl* |
| *Anthriscus sylvestris* subsp. *sylvestris* (L.) Hoffm. | #2638 | Poland, 50.9983°N 15.7601°E, 384 m, 22 June 2020, *P.Trzeciak & M.Piwczyński* (WA 0000165461) |  | PP110936 |  |  |  |  | *Syl* |
| *Anthriscus sylvestris* subsp. *sylvestris* (L.) Hoffm. | #2639 | Poland, 51.0332°N 15.8411°E, 314 m, 22 June 2020, *P.Trzeciak & M.Piwczyński* (WA 0000165462) |  | PP110937 |  |  |  |  | *Syl* |
| *Anthriscus sylvestris* subsp. *sylvestris* (L.) Hoffm. | #2640 | Poland, 51.1827°N 16.1023°E, 181 m, 22 June 2020, *P.Trzeciak & M.Piwczyński* (WA 0000165463) |  | PP110938 |  |  |  |  | *Syl* |
| *Anthriscus sylvestris* subsp. *sylvestris* (L.) Hoffm. | #2641 | Poland, 51.3959°N 16.2445°E, 137 m, 22 June 2020, *P.Trzeciak & M.Piwczyński* (WA 0000165464) |  | PP110939 |  |  |  |  | *Syl* |
| *Anthriscus sylvestris* subsp. *sylvestris* (L.) Hoffm. | #2642 | Poland, 51.6596°N 16.93°E, 106 m, 22 June 2020, *P.Trzeciak & M.Piwczyński* (WA 0000165465) |  | PP110940 |  |  |  |  | *Syl* |
| *Anthriscus sylvestris* subsp. *sylvestris* (L.) Hoffm. | #2643 | Poland, 49.4282°N 22.502°E, 499526978 m, 15 June 2021, *M.Piwczyński & P.Trzeciak* (WA 0000165466) |  | PP110952 |  |  |  |  | *Syl* |
| *Anthriscus sylvestris* subsp. *sylvestris* (L.) Hoffm. | #2644 | Poland, 49.3137°N 22.4355°E, 485347656 m, 15 June 2021, *M.Piwczyński & P.Trzeciak* (WA 0000165467) |  | PP110953 |  |  |  |  | *Syl* |
| *Anthriscus sylvestris* subsp. *sylvestris* (L.) Hoffm. | #2652 | Poland, 49.211°N 22.6811°E, 550716797 m, 15 June 2021, *M.Piwczyński & P.Trzeciak* (WA 0000165475) |  | PP110955 |  |  |  |  | *Syl* |
| *Anthriscus sylvestris* subsp. *sylvestris* (L.) Hoffm. | #2655 | Poland, 49.4583°N 22.6435°E, 425265625 m, 16 June 2021, *M.Piwczyński & P.Trzeciak* (WA 0000165478) |  | PP110956 |  |  |  |  | *Syl* |
| *Anthriscus sylvestris* subsp. *sylvestris* (L.) Hoffm. | #2658 | Poland, 49.5484°N 22.6066°E, 561050781 m, 16 June 2021, *M.Piwczyński & P.Trzeciak* (WA 0000165481) |  | PP110957 |  |  |  |  | *Syl* |
| *Anthriscus sylvestris* subsp. *sylvestris* (L.) Hoffm. | #2660 | Poland, 49.6543°N 22.6891°E, 312 m, 16 June 2021, *M.Piwczyński & P.Trzeciak* (WA 0000165483) |  | PP110958 |  |  |  |  | *Syl* |
| *Anthriscus sylvestris* subsp. *sylvestris* (L.) Hoffm. | #2661 | Poland, 49.623°N 22.7073°E, 439 m, 16 June 2021, *M.Piwczyński & P.Trzeciak* (WA 0000165484) |  | PP110959 |  |  |  |  | *Syl* |
| *Anthriscus sylvestris* subsp. *sylvestris* (L.) Hoffm. | #2662 | Poland, 49.6229°N 22.7069°E, 435 m, 16 June 2021, *M.Piwczyński & P.Trzeciak* (WA 0000165485) |  | PP110960 |  |  |  |  | *Syl* |
| *Anthriscus sylvestris* subsp. *sylvestris* (L.) Hoffm. | #2664 | Poland, 49.5421°N 21.6193°E, 393 m, 17 June 2021, *M.Piwczyński & P.Trzeciak* (WA 0000165487) |  | PP110961 |  |  |  |  | *Syl* |
| *Anthriscus sylvestris* subsp. *sylvestris* (L.) Hoffm. | #2667 | Poland, 49.4984°N 21.4218°E, 421 m, 17 June 2021, *M.Piwczyński & P.Trzeciak* (WA 0000165490) |  | PP110962 |  |  |  |  | *Syl* |
| *Anthriscus sylvestris* subsp. *sylvestris* (L.) Hoffm. | #2679 | Poland, 50.548°N 16.4299°E, 438 m, 26 June 2021, *M.Piwczyński* (WA 0000165502) |  | PP110963 |  |  |  |  | *Syl* |
| *Anthriscus sylvestris* subsp. *sylvestris* (L.) Hoffm. | #2685 | Poland, 50.5513°N 16.6608°E, 509 m, 27 June 2021, *M.Piwczyński* (WA 0000165508) |  | PP110964 |  |  |  |  | *Syl* |
| *Anthriscus tenerrima* Boiss. & Spruner | #0079 | Greece, Peloponnese region, Argolis regional unit, municipality Ermionida, NE Dhidhima, 820-880 m, 6 April 1979, *W.Greuter & H.Merxmüller* *16935* (E E00183143) | 37.50 23.21 1.027 km | ON180546 | ON584008 | ON584062 | ON637405 | ON637303 |  |
| *Anthriscus tenerrima* Boiss. & Spruner | #1211 | Greece, Central Greece region, Aetolia-Acarnania regional unit, Aktio-Vonitsa municipality, 2 km from the village of Kouvaras along main road to Rivio, 38°42′N 21°13′E, 20 m, 18 April 1989, *T.Landström & A.Strid* *28174* (G 458853) | 38.70 21.22 1.027 km | ON180547 |  | ON584063 | ON637406 | ON637304 |  |
| *Kozlovia capnoides* (Decne.) Spalik, Wojew. & S.R.Downie | #0216 | India, Uttarkhand state, Dehradun distr., near Koti Kanasar, 25 April 1894, *J.F.Duthie* *14473* (E E00040955) | 30.78 77.83 1.364 km | ON180527 |  | ON584058 | ON637400 | ON637298 |  |
| *Kozlovia laseroides* (Hedge & Lamond) Spalik & S.R.Downie | #0217 | Afghanistan, Tang-e Ghārō, E of Kabul, 1500 m, 2 April 1969, *H.Freitag* *4670* (W) | 34.56 69.51 0.702 km | ON180528 |  | ON584057 | ON637399 | ON637297 |  |
| *Kozlovia paleacea* (Regel & Schmalh.) Lipsky | #0218 | Afghanistan, Kataghan, village Pāygah Kowtal, between Pul-I Khumri and Haibak., 1500 m, 5 May 1967, *K.H.Rechinger* *33878* (E E00023059) | 36.19 68.30 50.000 km | ON180526 |  | ON584056 | ON637398 | ON637296 |  |

Supplementary Table S2. Characteristics of the datasets used in this study.

|  | ITS | partial *waxy* | *rpoB-trnC* spacer | *trnS–trnG* spacer | *psbA–trnH*  spacer | pDNA combined |
| --- | --- | --- | --- | --- | --- | --- |
| No. of sequences |  |  |  |  |  |  |
| all | 296 | 51 | 101 | 101 | 101 | 101 |
| unique | 58 | 32 | 63 | 63 | 63 | 63 |
| No. of aligned positions |  |  |  |  |  |  |
| total | 617 | 596 | 1272 | 582 | 173 | 2027 |
| constant | 475 | 522 | 1163 | 532 | 152 | 1847 |
| variable |  |  |  |  |  |  |
| parsimony-uninformative | 48 | 37 | 28 | 13 | 5 | 46 |
| parsimony-informative | 94 | 37 | 81 | 37 | 16 | 134 |
| excluded | 0 | 0 | 0 | 0 | 12 | 12 |

**Figure S1.** (A) Maximum likelihood tree obtained from ITS data. Numbers along branches indicate bootstrap support and/or posterior probability for corresponding nodes in Bayesian analyses. Only nodes with BS > 50 and PP > 0.5 are annotated.

**Figure S1.** (B) Bayesian tree obtained with MrBayes from ITS data. Numbers along branches indicate posterior probability. Only nodes with PP > 0.5 are annotated.

**Figure S2.** (A) Maximum likelihood tree obtained from waxy data. Numbers along branches indicate bootstrap support and/or posterior probability for corresponding nodes in Bayesian analyses. Only nodes with BS > 50 and PP > 0.5 are annotated.

**Figure S2**. (B) Bayesian tree obtained with MrBayes from *waxy* data. Numbers along branches indicate posterior probability. Only nodes with PP > 0.5 are annotated.

**Figure S3.** Bayesian tree obtained with MrBayes from plastid data. Numbers along branches indicate posterior probability. Only nodes with PP > 0.5 are annotated.
